# Supplementary material for: Prevalence of prolonged transitional neonatal hypoglycemia and associated factors in Ethiopia: A systematic review and meta-analysis
Source: PLoS One. 2025 Feb 6;20(2):e0316464. doi: 10.1371/journal.pone.0316464 (PMC11801580; doi:10.1371/journal.pone.0316464)
Supplement: S2 File — (DOCX) [file pone.0316464.s002.DOCX]

Supplementary file 2: JBI quality appraisal criteria

| Author | Criteria | | | | | | | | | Score | Quality |
| --- | --- | --- | --- | --- | --- | --- | --- | --- | --- | --- | --- |
|  | Was The Sample Frame Appropriate? | Was Sampling Appropriate? | Was The Sample Size Adequate? | Were The Study Subjects And The Setting Described In Detail? | Was The Data Analysis Conducted With Sufficient Coverage Of The Identified Sample? | Were Valid Methods Used For The Identification Of The Condition? | Was The Condition Measured In A Standard, Reliable Way For All Participants? | Was There Appropriate Statistical Analysis? | Was The Response Rate Adequate, And If Not, Was The Low Response Rate Managed Appropriately? |  |  |
| Sertsu Et Al., 2022 | Yes | Yes | Yes | Yes | Yes | Yes | Yes | Yes | Yes | 9 | Low risk |
| Fantahun & Nurussen, 2021 | yes | yes | no | yes | no | yes | yes | yes | no | 6 | Low risk |
| Chanie Et Al., 2023 | yes | yes | yes | no | yes | yes | no | yes | yes | 7 | Low risk |
| Demis Et Al., 2022 | yes | yes | yes | yes | yes | yes | yes | yes | yes | 9 | Low risk |
| Demisse Et Al., 2017 | yes | yes | yes | yes | not clear | yes | yes | yes | yes | 8 | Low risk |
| **Yohannes, 2021** | yes | yes | yes | not clear | yes | yes | yes | yes | yes | 8 | Low risk |
| Bogale Et Al., 2021 | yes | yes | yes | yes | yes | yes | yes | yes | yes | 9 | Low risk |
| Kassaye, 2021 | yes | yes | yes | yes | yes | yes | no | yes | no | 6 | Low risk |
