# Supplementary material for: Prevalence of prolonged transitional neonatal hypoglycemia and associated factors in Ethiopia: A systematic review and meta-analysis
Source: PLoS One. 2025 Feb 6;20(2):e0316464. doi: 10.1371/journal.pone.0316464 (PMC11801580; doi:10.1371/journal.pone.0316464)
Supplement: S4 File — (DOCX) [file pone.0316464.s004.docx]

**Supplementary file 4:** Studies identified in the literature search

| **Study No.** | **Author(s), Year** | **Title** | **Included/Excluded** | **Reason for Exclusion (if applicable)** |
| --- | --- | --- | --- | --- |
| 1 | Abramowski et al., 2021 | Neonatal hypoglycemia | Excluded | Summary text; no primary data |
| 2 | Agardh et al., 1992 | The influence of hypothermia on hypoglycemia-induced brain damage in the rat | Excluded | Animal study, not applicable to human neonatal hypoglycemia. |
| 3 | Alemu et al., 2017 | Neonatal hypoglycemia in diabetic mothers: A systematic review | Excluded | Specific to diabetic mothers, and systematic review, not original research. |
| 4 | Bromiker et al., 2019 | Early neonatal hypoglycemia: Incidence and risk factors | Excluded | Study out of Ethiopia, and different study design (cohort study). |
| 5 | Chanie et al., 2023 | Maximizing the detection rate of hypoglycemia among preterm neonates in Ethiopia | Included | N/A |
| 6 | Cornblath et al., 2000 | Controversies regarding the definition of neonatal hypoglycemia | Excluded | Not focused on original data for systematic review/meta-analysis |
| 7 | Demisse et al., 2017 | Patterns of admission and factors associated with neonatal mortality in Ethiopia | Included | N/A |
| 8 | Ekubay et al., 2018 | Initiation of breastfeeding within one hour of birth in Addis Ababa, Ethiopia | Excluded | Not related to neonatal hypoglycemia or feeding intolerance. |
| 9 | Ellahony et al., 2020 | Prevalence of glucose level abnormalities in neonatal sepsis | Excluded | Focuses on neonatal sepsis rather than neonatal hypoglycemia or feeding intolerance. |
| 10 | Kasaye et al., 2021 | Prevalence and predictors of hypoglycemia in neonates in Addis Ababa, Ethiopia | Included | N/A |
| 11 | Frank-Briggs et al., 2008 | Neonatal hypoglycemia: Prevalence and clinical manifestations in Nigeria | Excluded | Data outside the geographic context of interest |
| 12 | Gebremichael et al., 2020 | Under-five mortality and associated risk factors in rural settings of Ethiopia | Excluded | Does not address neonatal hypoglycemia or feeding intolerance. |
| 13 | Goode et al., 2016 | Developmental outcomes of preterm infants with neonatal hypoglycemia | Excluded | Focused on preterm infants with hypoglycemia. |
| 14 | He et al., 2022 | Association between neonatal hyperbilirubinemia and hypoglycemia in China | Excluded | Not relevant to neonatal hypoglycemia. |
| 15 | Ioannidis et al., 2008 | Interpretation of tests of heterogeneity and bias in meta-analysis | Excluded | Methodological focus, not clinical neonatal research. |
| 16 | Islam et al., 2017 | Glycemic status and its effect in neonatal sepsis in a tertiary care hospital | Excluded | Focuses on neonatal sepsis rather than specifically on neonatal hypoglycemia or feeding intolerance. |
| 17 | Harding et al., 2024 | Neonatal hypoglycemia | Excluded | Not directly related to Ethiopian population or study context. |
| 18 | Kallem et al., 2020 | Infant of diabetic mother: What one needs to know? | Excluded | Focus on infants of diabetic mothers, not general neonatal population. |
| 19 | Karim et al., 2018 | Initiation of breastfeeding within one hour of birth in Bangladesh | Excluded | Focuses on breastfeeding initiation, not on neonatal hypoglycemia or feeding intolerance. |
| 20 | Kallem et al., 2020 | Infant of diabetic mother: what one needs to know | Excluded | Focus on infants of diabetic mothers, not general neonatal population. |
| 21 | Karim et al., 2018 | Initiation of breastfeeding within one hour of birth and its determinants among normal vaginal deliveries at primary and secondary health facilities in Bangladesh: A case-observation study | Excluded | Focuses on breastfeeding initiation, not on neonatal hypoglycemia or feeding intolerance. |
| 22 | Kedes & Field, 1964 | Hypothermia. New England Journal of Medicine | Excluded | Not related to hypoglycemia or human studies. |
| 23 | Khaydarov et al., 2023 | Heart failure and the risk of hypoglycemia. Science and Education | Excluded | Focuses on the relationship between heart failure and hypoglycemia, not neonatal hypoglycemia. |
| 24 | Mormile, 2016 | Neonates of diabetic mothers: The starting point for developing novel therapeutic approaches to ischemic heart and brain? Medical Hypotheses | Excluded | Not directly related to neonatal hypoglycemia. |
| 25 | Najati & Saboktakin, 2010 | Prevalence and underlying etiologies of neonatal hypoglycemia. Pakistan Journal of Biological Sciences | Excluded | Study conducted in a different population group (Pakistan). |
| 26 | Nurussen & Fantahun, 2021 | Prevalence and Risk Factors of Neonatal Hypoglycemia at St. Paul’s Hospital Millennium Medical College, Ethiopia | Included | N/A |
| 27 | O’Brien et al., 2023 | Infants eligible for neonatal hypoglycemia screening: A systematic review | Excluded | Does not include data related to neonatal hypoglycemia management or interventions. |
| 28 | Opara et al., 2010 | Morbidity and mortality amongst infants of diabetic mothers admitted into a special care baby unit in Port Harcourt, Nigeria | Excluded | Focus on infants of diabetic mothers. |
| 29 | Paul & Fantahun, 2020 | Prevalence and risk factors of hypoglycemia in neonates: A cross-sectional study | Excluded | Overlapping data with another included study. |
| 30 | Peters et al., 2015 | The Joanna Briggs Institute reviewers’ manual 2015 | Excluded | Methodological guide, not a primary research study. |
| 31 | Rop et al., 2023 | Prevalence of neonatal hypoglycemia and risk factors in the newborn unit at a tertiary care hospital in Kenya. East African Medical Journal, 100(4), 5838-5832. | Excluded | Study focused on a specific hospital in Kenya, and the data does not align with the geographical scope of the research. |
| 32 | Salman et al., 2021 | Frequency of immediate neonatal complications (hypoglycemia and neonatal jaundice) in late preterm and term neonates | Excluded | Directly not relevant to neonatal hypoglycemia and feeding issues. |
| 33 | Sertsu et al., 2022 | Determinants of neonatal hypoglycemia among neonates admitted at Hiwot Fana Comprehensive Specialized University Hospital, Eastern Ethiopia: A retrospective cross-sectional study | Included | N/A |
| 34 | Sharma et al., 2017 | Hypoglycemia in the preterm neonate: Etiopathogenesis, diagnosis, management, and long-term outcomes. | Excluded | Focus on preterm neonates, not applicable to full-term infants. |
| 35 | Demis et al., 2022 | Incidence of Persistent Neonatal Hypoglycemia and Associated Factors among Neonatal Intensive Care Unit Admissions in South Gondar Public Hospitals, North-central Ethiopia: a prospective cross-sectional study | Included | N/A |
| 36 | Stanley et al., 2023 | New approaches to screening and management of neonatal hypoglycemia based on improved understanding of the molecular mechanism of hypoglycemia | Excluded | Focuses on molecular mechanisms rather than screening or management approaches. |
| 37 | Sweet et al., 2013 | Management strategies for neonatal hypoglycemia. | Excluded | Relevant to neonatal hypoglycemia management, analysis. |
| 38 | Teshome & Akanaw, 2021 | Factors Associated with Neonatal Hyperbilirubinemia in Case Files of All Admitted Inborn and Outborn Neonates in Northwest Ethiopia. | Included | N/A |
| 39 | Thompson-Havranek, 2017 | Neonatal hypoglycemia | Excluded | Review article. |
| 40 | Tran et al., 2012 | Hypothermia is a frequent sign of severe hypoglycemia in patients with diabetes. Diabetes & Metabolism | Excluded | Focus on diabetes in adults, not neonatal hypoglycemia. |
| 41 | Voormolen et al., 2018 | Neonatal hypoglycemia following diet-controlled and insulin-treated gestational diabetes mellitus. | Excluded | Focuses on neonatal hypoglycemia in a specific group (gestational diabetes), not broadly applicable. |
| 42 | World Health Organization (WHO), 2017 | Guideline: Protecting, promoting, and supporting breastfeeding in facilities providing maternity and newborn services | Excluded | Guidelines and not primary research. |
| 43 | Yitayew et al., 2020 | Neonatal hypothermia and associated factors among newborns admitted in the neonatal intensive care unit of Dessie Referral Hospital, Amhara Region, Northeast Ethiopia. | Excluded | Focus on neonatal hypothermia, not hypoglycemia. |
| 44 | Yohannes et al., 2021 | Magnitude of Neonatal Jaundice and Its Associated Factors among Neonates Admitted to Neonatal Intensive Care Unit in Selected Public Hospitals at Harar and Dire Dawa, Eastern Ethiopia | Included | N/A |
| 45 | You et al., 2015 | Global, regional, and national levels and trends in under-5 mortality between 1990 and 2015 | Excluded | Focus on under-5 mortality, not neonatal outcomes or hypoglycemia. |
| 46 | Zhou et al., 2015 | Hypoglycemia incidence and risk factors assessment in hospitalized neonates | Excluded | Study focused on neonatal hypoglycemia assessment. |
