# Supplementary material for: Prevalence of prolonged transitional neonatal hypoglycemia and associated factors in Ethiopia: A systematic review and meta-analysis
Source: PLoS One. 2025 Feb 6;20(2):e0316464. doi: 10.1371/journal.pone.0316464 (PMC11801580; doi:10.1371/journal.pone.0316464)
Supplement: Attachment — Submitted filename: PONE-D-23-19848.pdf [file pone.0316464.s006.pdf]

PLOS ONE

Prevalence of persistent neonatal hypoglycemia and its associated factors in Ethiopia: A systematic review and meta-analysis

--Manuscript Draft--

Manuscript Number: PONE-D-23-19848

Article Type: Research Article

Full Title: Prevalence of persistent neonatal hypoglycemia and its associated factors in Ethiopia: A systematic review and meta-analysis

Short Title: Prevalence of persistent neonatal hypoglycemia and its associated factors in Ethiopia: A systematic review

Corresponding Author: Solomon Demis
Debre Tabor University
Debre Tabor, South Gondar ETHIOPIA

Keywords: prevalence, persistent neonatal hypoglycemia, prolonged neonatal hypoglycemia, Ethiopia

Abstract:

Objectives: persistent neonatal hypoglycemia is the severe form of neonatal hypoglycemia with neurodevelopmental consequences. Decreasing preventable neonatal death is an essential public health concern for the resource-limited country including Ethiopia. Thus, the pooled persistent neonatal hypoglycemia which can be prevented and associated factors was identified.

Design: A weighted inverse-variance random effects model was used to compute the overall pooled prevalence and associated factors. Variations across the studies were checked using the forest plot, I2 statistics, and Egger's test.

Setting: National neonatal intensive care units in Ethiopia

Participants: 8 primary studies with a sample size of 3686 neonates.

Outcome measures: the neonates with random blood sugar (RBS) <47 mg/dL after 48-72 hours of age after delivery at bedside glucose check from a capillary sample.

Results: a total of 8 studies with a sample of 3686 neonates were included in this meta-analysis. The pooled estimate of persistent neonatal hypoglycemia (PNHG) in Ethiopia was 19.71% (95% CI: 16.85-22.56); I2=79.20, P<0.001)). A sample size of > 400 and ≤ 400 was found to be comparable at 18% (15-22) and 21% (17-26) respectively. On the other hand, using the RBS cutoff <47 mg/dL and <40 mg/dL, PNHG was relatively similar at 21% (16-27) and 18%(15-22) respectively. Neonates delivered prematurely (AOR=3.31; 95% CI: 2.57-4.04), hypothermia (AOR=3.41; 95%CI: 2.19-4.62), IDM (AOR=4.71; 95%CI: 2.15-7.26), breastfeeding initiation after an hour (AOR=3.26; 95% CI: 2.03-4.49), pathologic jaundice (AOR=2.37; 95%CI: 1.91-2.84) were significantly associated with PNHG.

Conclusion: it was found that nearly one-fifth of the neonates in Ethiopia were in a state of hypoglycemia during their treatment regimen. Fortunately, the factors associated with PNHG were easily modifiable in the healthcare setup. Hence, prioritization of the problem in current national NICU hospital practice guidelines, updating hypoglycemia prevention modalities including initiation of early breastfeeding particularly for those neonates delivered by cesarean section, early intervening on factors associated with, training about the disorder and its impact on neonates would reduce the burden in Ethiopia.

Prospero ID: CRD42023424953.

Order of Authors:

Solomon Demis
Shegaw Zeleke
Amare kasaw
Tigabu Munye
Yohannes Tesfahun
Amare Simegn
Worku Necho

Powered by Editorial Manager® and ProduXion Manager® from Aries Systems Corporation

	Kindu Agmas
	Demewoz Kefale
**Additional Information:**	
**Question**	**Response**
**Financial Disclosure**<br><br>Enter a financial disclosure statement that describes the sources of funding for the work included in this submission. Review the submission guidelines for detailed requirements. View published research articles from *PLOS ONE* for specific examples.<br><br>This statement is required for submission and **will appear in the published article** if the submission is accepted. Please make sure it is accurate.<br><br>**Unfunded studies**<br>Enter: *The author(s) received no specific funding for this work.*<br><br>**Funded studies**<br>Enter a statement with the following details:<br>• Initials of the authors who received each award<br>• Grant numbers awarded to each author<br>• The full name of each funder<br>• URL of each funder website<br>• Did the sponsors or funders play any role in the study design, data collection and analysis, decision to publish, or preparation of the manuscript?<br>• **NO** - Include this sentence at the end of your statement: *The funders had no role in study design, data collection and analysis, decision to publish, or preparation of the manuscript.*<br>• **YES** - Specify the role(s) played.<br><br>* typeset	No
**Competing Interests**<br><br>Use the instructions below to enter a competing interest statement for this submission. On behalf of all authors, disclose any competing interests that could be perceived to bias this work—acknowledging all financial support and any other relevant financial or non-	No authors have competing interests.

financial competing interests.<br><br>This statement is required for submission and **will appear in the published article** if the submission is accepted. Please make sure it is accurate and that any funding sources listed in your Funding Information later in the submission form are also declared in your Financial Disclosure statement.<br><br>View published research articles from *PLOS ONE* for specific examples.<br><br>**NO authors have competing interests**<br><br>Enter: *The authors have declared that no competing interests exist.*<br><br>**Authors with competing interests**<br><br>Enter competing interest details beginning with this statement:<br><br>*I have read the journal's policy and the authors of this manuscript have the following competing interests: [insert competing interests here]*<br><br>* typeset	
**Ethics Statement**<br><br>Enter an ethics statement for this submission. This statement is required if the study involved:<br><br>• Human participants<br>• Human specimens or tissue<br>• Vertebrate animals or cephalopods<br>• Vertebrate embryos or tissues<br>• Field research<br><br>Write "N/A" if the submission does not require an ethics statement.<br><br>General guidance is provided below.<br>Consult the submission guidelines for	Not applicable.

detailed instructions. **Make sure that all information entered here is included in the Methods section of the manuscript.**<br><br>**Format for specific study types**<br><br>**Human Subject Research (involving human participants and/or tissue)**<br>• Give the name of the institutional review board or ethics committee that approved the study<br>• Include the approval number and/or a statement indicating approval of this research<br>• Indicate the form of consent obtained (written/oral) or the reason that consent was not obtained (e.g. the data were analyzed anonymously)<br><br>**Animal Research (involving vertebrate animals, embryos or tissues)**<br>• Provide the name of the Institutional Animal Care and Use Committee (IACUC) or other relevant ethics board that reviewed the study protocol, and indicate whether they approved this research or granted a formal waiver of ethical approval<br>• Include an approval number if one was obtained<br>• If the study involved *non-human primates*, add *additional details* about animal welfare and steps taken to ameliorate suffering<br>• If anesthesia, euthanasia, or any kind of animal sacrifice is part of the study, include briefly which substances and/or methods were applied<br><br>**Field Research**<br><br>Include the following details if this study involves the collection of plant, animal, or other materials from a natural setting:<br>• Field permit number<br>• Name of the institution or relevant body that granted permission	
**Data Availability**<br><br>Authors are required to make all data underlying the findings described fully available, without restriction, and from the time of publication. PLOS allows rare	Yes - all data are fully available without restriction

exceptions to address legal and ethical concerns. See the PLOS Data Policy and FAQ for detailed information.<br><br>A Data Availability Statement describing where the data can be found is required at submission. Your answers to this question constitute the Data Availability Statement and **will be published in the article**, if accepted.<br><br>**Important:** Stating 'data available on request from the author' is not sufficient. If your data are only available upon request, select 'No' for the first question and explain your exceptional situation in the text box.<br><br>Do the authors confirm that all data underlying the findings described in their manuscript are fully available without restriction?	
**Describe where the data may be found in full sentences. If you are copying our sample text, replace any instances of XXX with the appropriate details.**<br><br>• If the data are **held or will be held in a public repository**, include URLs, accession numbers or DOIs. If this information will only be available after acceptance, indicate this by ticking the box below. For example: *All XXX files are available from the XXX database (accession number(s) XXX, XXX.*).<br>• If the data are all contained **within the manuscript and/or Supporting Information files**, enter the following: *All relevant data are within the manuscript and its Supporting Information files.*<br>• If neither of these applies but you are able to provide **details of access elsewhere**, with or without limitations, please do so. For example:<br><br>*Data cannot be shared publicly because of [XXX]. Data are available from the XXX Institutional Data Access / Ethics Committee (contact via XXX) for researchers who meet the criteria for*	The data will be available upon reasonable request.

access to confidential data.<br><br>*The data underlying the results presented in the study are available from (include the name of the third party and contact information or URL).*<br>• This text is appropriate if the data are owned by a third party and authors do not have permission to share the data.<br><br>* typeset	
Additional data availability information:	Tick here if your circumstances are not covered by the questions above and you need the journal's help to make your data available.

Powered by Editorial Manager® and ProduXion Manager® from Aries Systems Corporation

Manuscript
Click here to access/download;Manuscript;PNHG SR MA PONE.docx

Prevalence of persistent neonatal hypoglycemia and its associated factors in Ethiopia: A systematic review and meta-analysis

Solomon Demis[1*], Shegaw Zeleke[2], Amare Kasaw[3], Tigabu Munye[4], Yohannes Tesfahun[5], Amare Simegn Ayele[6], Worku Necho[7],Kindu Agmas[8], Demewoz Kefale[9]

1* Department of Maternity and neonatal nursing, College of health sciences, Debre Tabor University, Ethiopia

2, 4 Department of Nursing, College of health sciences, Debre Tabor University, Ethiopia

3, 9 Department of Pediatrics and child health nursing, College of health sciences, Debre Tabor University, Ethiopia

5 Department of Emergency and critical care, College of health sciences, Debre Tabor University, Ethiopia

6 Department of Midwifery, college of health sciences, Debre Tabor University, Ethiopia

7 Department of Maternity and neonatal nursing, College of health sciences, Debre Tabor University, Ethiopia

8 Department of Pediatrics and child health, Debre Tabor Comprehensive Specialized Hospital, Ethiopia

Corresponding author: Solomon Demis, email: solomondemis@gmail.com, mob. +251930713008

Abstract

Objectives: persistent neonatal hypoglycemia is the severe form of neonatal hypoglycemia with neurodevelopmental consequences. Decreasing preventable neonatal death is an essential public health concern for the resource-limited country including Ethiopia. Thus, the pooled persistent neonatal hypoglycemia which can be prevented and associated factors was identified.

Design: A weighted inverse-variance random effects model was used to compute the overall pooled prevalence and associated factors. Variations across the studies were checked using the forest plot, I^2 statistics, and Egger's test.

Setting: National neonatal intensive care units in Ethiopia

Participants: 8 primary studies with a sample size of 3686 neonates.

Outcome measures: the neonates with random blood sugar (RBS) <47 mg/dL after 48-72 hours of age after delivery at bedside glucose check from a capillary sample.

Results: a total of 8 studies with a sample of 3686 neonates were included in this meta-analysis. The pooled estimate of persistent neonatal hypoglycemia (PNHG) in Ethiopia was 19.71% (95% CI: 16.85-22.56); I2=79.20, P<0.001)). A sample size of > 400 and ≤ 400 was found to be comparable at 18% (15-22) and 21% (17-26) respectively. On the other hand, using the RBS cutoff <47 mg/dL and <40 mg/dL, PNHG was relatively similar at 21% (16-27) and 18%(15-22) respectively. Neonates delivered prematurely (AOR=3.31; 95% CI: 2.57-4.04), hypothermia (AOR=3.41; 95%CI: 2.19-4.62), IDM (AOR=4.71; 95%CI: 2.15-7.26), breastfeeding initiation after an hour (AOR=3.26; 95% CI: 2.03-4.49), pathologic jaundice (AOR=2.37; 95%CI: 1.91-2.84) were significantly associated with PNHG.

Conclusion: it was found that nearly one-fifth of the neonates in Ethiopia were in a state of hypoglycemia during their treatment regimen. Fortunately, the factors associated with PNHG were easily modifiable in the healthcare setup. Hence, prioritization of the problem in current national NICU hospital practice guidelines, updating hypoglycemia prevention modalities including initiation of early breastfeeding particularly for those neonates delivered by cesarean section, early intervening on factors associated with, training about the disorder and its impact on neonates would reduce the burden in Ethiopia.

Prospero ID: CRD42023424953.

Keywords: prevalence, persistent neonatal hypoglycemia, prolonged neonatal hypoglycemia, Ethiopia

Background

As the World Health Organization (WHO) and American Academy of Pediatrics (AAP) defined the term neonatal hypoglycemia is the low blood glucose concentration from the population as a blood glucose level below 40-50 mg/dL. Nearly at the same level, the Ethiopian hospital's intensive care unit (NICU) practice guideline defined neonatal hypoglycemia as a blood glucose level less than 40 gm/dL which requires further evaluation and intervention (1–4).

There are two types of neonatal hypoglycemia, transient and persistent. Most neonates have transient hypoglycemia which responds to treatment and is associated with a good prognosis compared with persistent hypoglycemia. If hypoglycemia occurs after 48 hours of age is termed persistent pathological hypoglycemia by which clinical manifestations are generated in the short term with abrupt injury and neurodevelopmental sequelae in long-term effect (3,4).

[illegible] normal physiology, the neonates' random blood glucose level (RBS) drops by 25–30 mg/dL and may lower down to 55–60 mg/dL in the first three hours of life. In the normal transition to extrauterine life, the glucose levels then steadily rise over the first few days of life with the help of different adaptation mechanisms. With the sudden discontinuation of the placental supply of nutrients after birth, the neonates engaged in the adaptive process including the mobilization of glucose and fatty acids(1,3,4).

Glucose is the main source of fuel for the brain. The glucose requirement of the brain is about 90% of the total glucose requirement in neonates. That is why newborns need to maintain regular and more frequent feedings by the first few days after birth to maintain their brains intact and developing. Since the neonatal gut is immature, metabolizing of feeding at a rate that supports the production and maintenance of normal blood glucose concentrations may lead to hypoglycemia(5–7).

Deviations from the normal transition can occur as a result of a wide range of factors predisposing to neonatal hypoglycemia. The brain uses glucose primarily to meet its metabolic requirements. Thus, the main mechanisms by which these factors result in hypoglycemia include disruption of gluconeogenesis, decreased alternate fuel production, increased glucose demand, and failure to receive or absorb nutrients. That is why neonatal hypoglycemia is one of the significant medical emergencies in a neonatal unit. The signs and symptoms are often non-specific. Some are completely asymptomatic. Many cases, therefore, remain undiagnosed. The diagnosis of neonatal hypoglycemia requires a high index of suspicion since its presentation is different. Early diagnosis and treatment could prevent abrupt signs and symptoms, long-term complications, and death (1,2,8).

The reason that neonatal hypoglycemia is still a severe concern is that it causes neurological sequelae, increased risk of mortality, and other complications to the neonate and the family. The study conducted by Rajesh S., et al 2019, revealed that 8% of neonates with persistent hypoglycemia had cerebral palsy, Odds of epilepsy among hypoglycemic neonates were 1.93 and increases the risk of the specific cognitive defect by 2-3 folds in early childhood. Similarly, in late childhood development, neonatal hypoglycemia had odds of 3.2 for neurodevelopmental impairment and 2.04 for low literacy at their school (9).

Neonates with persistent neonatal hypoglycemia had an additional burden of increased risk for mortality. Studies in Tanzania showed that hypoglycemia was found to be a major cause of neonatal mortality, contributing to 20% of the causes. Other family-related burdens are secondary to hypoglycemia neonatal admission were increased costs to the family and separation from the mother(10). The Women and child health section of WHO recommends that preterm and those small for gestational age (SGA) neonates show a constrained blood glucose regulatory mechanism to hypoglycemia. Thus, detection and treatment in these groups of neonates are targeted to reduce neonatal death mainly in low-resource countries (11–14).

Random blood glucose level (RBS) values below 47 mg/dL (2.6 mmol/L), If present after 48 hours during the first two months of life, showed a significant statistical correlation with acute seizure activities, abnormal neuromotor manifestations, and impaired intellectual performance at 18 months of age(1,3,4).

The reported prevalence of hypoglycemia varies from center to center depending on the cutoff values of blood glucose for the diagnosis of hypoglycemia. Neonatal hypoglycemia affects up to about 4-5 per 1000 term infants according to some studies (2,8).

Despite progress over the past two decades, the age distribution of the mortality of children and young adolescents shows that the highest risk of death is during the neonatal period (the first 28 days of life) with preventable causes which accounts for 2.5 million out of 6.2 million death of children and young adolescents in 2018 alone. The neonatal mortality rate was estimated at 18 deaths per 1,000 live births globally. The probability of dying after the first month and before reaching age 1 was 11 per 1,000 live births (4,15).

Neonatal hypoglycemia (NHG) is an under-recognized cause of morbidity and mortality. In some studies, the incidence of NHG varies due to different factors as a result it cannot be estimated easily by other countries finding (1,16).

According to the Ethiopian Demographic health survey (EDHS), Ethiopia has one of the highest neonatal mortality rates than global rates which was accounted for (29 per 1000 live births), Which has poor progress in reducing the rate of neonatal mortality from 2000 to 2016 according to EDHS 2016. Decreasing preventable neonatal death is an essential public health concern for the resource-limited country of Ethiopia(17).

Previous studies on the incidence of neonatal hypoglycemia and its risk factors are limited and there is no review on persistent neonatal hypoglycemia. And, the increased prevalence of prematurity and low birth weight makes such review vital to the formulation of recommendations for prevention and management to reduce neonatal mortality in specific and under-five children in general.

Methods and materials

The results of this review were reported based on the Preferred Reporting Items for Systematic Review and Meta-analysis (PRISMA) statement guideline(18) (**supplementary file 1**), and it is registered in the PROSPERO database (PROSPERO ID:).

Search strategy and information source

The adapted PICO format was used to explicitly review the pieces of literature and clear specifications for the inclusion and exclusion criteria. The adapted PICO comprises Population (P), Exposure (E), Outcome (O), and Context (Setting as described below.

a. **Population**: preterm neonates
b. **Exposure**: Associated factors, predictors, risk factors
c. **[illegible]tcome**: Intraventricular Hemorrhage
d. **Context (Setting):** countries found in Africa.

Hence, by using this adapted PICO format, the following review questions were developed for the search for data.

1. [illegible]at is the magnitude of IVH in African countries?
2. What are the predictors of IVH in African countries?

Based on the aforementioned review PICO format and questions, primary studies searching was done from MEDLINE, Science Direct, Google Scholar, African Journal Online, and Addis Ababa University library repository were searched.

T[illegible] core search terms and phrases were “prevalence”, “incidence”, “epidemiology”, “proportion”, “magnitude”, “burden”, “Intraventricular” and ‘’predictors’’, “risk factors’’, ‘’associated factors’’ and ‘’Africa’’ Or ‘’African countries’’. The search strategies were developed using different Boolean operators. Notably, to fit the advanced MEDLINE database, the following search strategy was applied on May 12, 2023: [(prevalence[MeSH Terms]) OR incidence[MeSH Terms]) OR proportion [MeSH Terms]) OR epidemiology[MeSH Terms]) OR magnitude[MeSH Terms]) OR burden[MeSH Terms]) AND predictors [MeSH Terms]) OR risk factors [MeSH Terms]) OR associated factors [MeSH Terms]) OR neonatal intraventricular hemorrhage [MeSH Terms]) OR newborn intraventricular bleeding [MeSH Terms]) OR preterm intraventricular hemorrhage [MeSH Terms]) OR Cerebral intracranial hemorrhage [MeSH Terms]) AND (African countries)].

Study selection

Primary studies downloaded and retrieved studies were exported to the Mendeley Desktop 1.19.8 reference manager software to remove duplicate studies. The study selection process was held in

two stages. Firstly, the title and abstract were screened and secondly, full-text reviewing was followed. Two independent reviewers (DKM and SZB) screened the title and abstract. The disagreement was handled based on established article selection criteria and the third author (SDK) settled the disagreement to decide whether the study was eligible to be included or not. Then two independent authors conducted the abstract and full-text review for eligible articles.

Eligibility criteria

Inclusion criteria

Included studies were articles that reported the prevalence of persistent neonatal hypoglycemia in general and/or at least one associated factor. It also included studies published in English and studies conducted only in Ethiopia.

Exclusion criteria

Articles without full-text available and qualitative studies were excluded. Likewise, reviews, commentaries, consultants' corners, letters, and conference abstracts were not considered.

Critical appraisal and quality assessment

We used Joanna Briggs Institute's (JBI) quality appraisal criteria(19). The tool consists of nine major items. The first item is appropriate to the sample frame. The second is the appropriate sampling technique. The third is the adequacy of the sample size. The fourth is a description of the study subjects and settings. The fifth is enough coverage of data analysis. The sixth is the validity of the method for the identification of the condition. The seventh item is a standard and reliable measurement for all participants. The eighth is the appropriateness of statistical analysis. And the last item is adequacy and management of the response rate. Studies were considered low-risk when they would fit 5 or above quality assessment checklists. Two independent authors (TMA and WNA) appraised the quality of the studies. The disagreement was resolved by the interference of a third reviewer (SDK). The quality scores measuring the status of the primary study were reported (**Supplementary file 2**).

Data extraction

The data was determined to be extracted based on the two criteria namely: 1. Clear and consistent operational definition for the dependent variable (Persistent neonatal hypoglycemia) and 2. Those variables are statistically associated with the outcome variable reported by AOR. Two authors extracted data using the standardized format of MS Excel spreadsheet. The name of the first author and year, study region, study area, study design, Random blood glucose (RBS) cut point, sample size, prevalence, and odds of associated factors were extracted. Whenever variations are observed, the phase was repeated. If discrepancies between data extractors continued, the third reviewer was involved in the decision. Having faced reports of the inconsistency of data in the primary study, data transformation was conducted.

Outcome measurement

Diagnosis of Persistent neonatal hypoglycemia: the neonates with random blood sugar (RBS) <47 mg/dL after 48-72 hours of age after delivery at bedside glucose check from a capillary sample (3,4).

Statistical analysis

The required data were collected using a Microsoft Excel 2013 workbook. Then, the STATA version 17 statistical software was used for meta-analysis. Publication bias was objectively checked using Egger's regression test analysis(20). The heterogeneity of studies was quantified using the I-squared statistic (21). Both pooled prevalence analysis and pooled effect of associated factors were conducted using a weighted inverse variance random-effects model (22). Subgroup analysis was done by the diagnostic method and sample size to overcome the inflation of the pooled effect from the inclusion of studies done.

Results

Literature search

The search strategy retrieved 64 articles electronically and manually, of which 18 duplicate articles were identified and removed. Among screened articles 46, they were from PubMed (n=19), Google Scholar (n=23), and other databases of the Addis Ababa University repository

(n=2) and Haramaya University database (n=1), and manuscript (n=1). Nineteen articles were reviewed for full text and eight studies were included for review and meta-analysis (Fig.1).

Characteristics of included studies

A total of eight cross-sectional studies with a pooled sample size of 3,686 neonates were included in this meta-analysis. Two studies were found in each part of Ethiopia namely at Central, Eastern, Northwest and Northern respectively (2,23–29). Four studies used the cut point of RBS <40mg/dl for the diagnosis of persistent neonatal hypoglycemia (23,24,28,29). The remaining four studies used the RBS cut point of less than 47 mg/dl for the diagnosis of PNHG (2,26,27,30). The maximum sample size was 769 and the minimum was 196 among the reviewed studies (28,30). The studies included in this systematic review and meta-analysis had no considerable risk. Therefore, all the studies were considered. We assessed the studies with a JBI quality appraisal checklist for systematic review (31). As per this appraisal checklist, none of the included studies was of poor quality status with a quality score of ≥6 (**Table 1**).

Table 1: Characteristics and quality status of the studies in the review of prevalence and associated factors of PNHG in Ethiopia

Author	Study area	RBS cut point	Sample size	Prevalence	Quality status
Bogale et al., 2021	UOG	<40mg/dl	399	13.53	Low risk
Demis et al., 2022	DTCSH	<47mg/dl	400	23.50	Low risk
Fantahun & Nurussen, 2021	St. PHMMC	<47mg/dl	196	25.00	Low risk
Kasaye, 2021	Addis Ababa	<40mg/dl	317	20.82	Low risk
Sertsu et al., 2022	HFSTH	<40mg/dl	698	21.20	Low risk
Yohannes, 2021	Harar and Dire Dawa	<47mg/dl	640	14.68	Low risk
Chanie et al., 2023	DTCSH	<47mg/dl	267	23.60	Low risk

Demisse et al., 2017	UOG	<40mg/dl	769	18.46	Low risk

UOG=university of Gondar, St. PHMMC=Saint Paul's Millennium medical college, DTCSH=Debre tabor comprehensive specialized hospital

Meta-analysis

The pooled prevalence of persistent neonatal hypoglycemia

The absence of publication bias was assessed with Egger's regression test analysis (p = 0.068), which showed no publication bias (**Table 2**).

Table 2: Egger's test (Quantitative evidence of no publication bias)

**Std.-Eff.**	**Coef.**	**Std. Err**	**t**	**p>t**	**95% confidence interval**	
**Slope**	5.21962	2.177999	2.40	0.054	-.1097508	10.54899
**Bias**	9.567008	4.312786	2.22	**0.068**	-.9859986	20.12001

The pooled prevalence of PNHG from eight studies (Demisse *et al.*, 2017; Bogale *et al.*, 2021; Fantahun and Nurussen, 2021; Kasaye, 2021; Yohannes, 2021; Demis *et al.*, 2022; Sertsu *et al.*, 2022; Chanie *et al.*, 2023) was 19.71(95% CI: 16.85-22.56); I^2=79.20, P<0.001 (**fig 2**).

Subgroup analysis

Subgroup analysis was done by using the sample size, and the RBS cut point to diagnose persistent neonatal hypoglycemia. However, there was no effect in reducing their heterogeneity measurement (I^2). From the three studies of pooled effect, the prevalence of PNHG by studies with sample size >400 was found to be 18% (15-22) and the polled effect of the five studies with a sample size of <= 400 was 21% (17-26) (**fig 3**).

The subgroup analysis of pooled effect by RBS cutoff point for the diagnosis of PNHG, RBS cutoff point for the diagnosis of PNHG by <40mg/dl was 18%(15-22) whereas the RBS cutoff point by <47mg/dl was 21%(16-27)(**Fig.4**).

Analysis of factors associated with PNHG

Based on this review, the associated factors for persistent neonatal hypoglycemia by pooling two or more studies were presented as preterm birth, neonatal hypothermia, neonates from chronic diabetic mellitus mother, breastfeeding initiation after an hour, and pathological jaundice.

Neonates delivered prematurely by studies (24,27) were three more likely to develop persistent neonatal hypoglycemia compared to neonates with term birth in Ethiopia (AOR=3.31; 95% CI: 2.57-4.04). By pooled analysis of four studies (24,26,27,30) revealed that neonates with hypothermia were 3.41 times (AOR=3.41; 95%CI: 2.19-4.62) more likely to develop PNHG that their norm-thermic counterparts. Neonates whose mothers were known chronic DM were nearly five times(AOR=4.71; 95%CI: 2.15-7.26) than those whose mothers were not DM by studies (23,24,26). Regarding breastfeeding initiation; by studies (23,24), neonates who have been breastfeeding after an hour were three times more likely to be hypoglycemic than neonates breastfeeding within an hour(AOR=3.26; 95% CI:2.03-4.49). By pooling of two studies(25,27) revealed that pathologic jaundice of neonates was almost two and a half times (AOR=2.37; 95%CI: 1.91-2.84) more likely to be PNHG than their counterparts without pathological jaundice (**Table 3**).

Table 3: The pooled effect of factors associated with PNHG in Ethiopia, 2023.

Factors	Author and year	Pooled OR (95% CI)	I^2(P value)
Preterm birth	(24)	3.31(2.57-4.04)	0.00%(0.00)
	(27)		
Neonatal Hypothermia	(24)	3.41(2.19-4.62)	97.96%(0.00)
	(30)		
	(26)		
	(27)		
Neonates from chronic diabetic mother	(24)	4.71(2.15-7.26)	94%(0.00)
	(26)		
	(Kasaye, 2021)		
Breastfeeding	(24)	3.26(2.03-4.49)	52.80%(0.00)

after one hour	(Kasaye, 2021)		
Pathological jaundice	(27)	2.37(1.91-2.84)	0.00%(0.00)
	(Yohannes, 2021)		

The studies by (24) revealed that the odds of neonatal sepsis were nearly three times that of their counterparts with no sepsis and the odds of neonates with VLBW were four times than neonates with NBW at (AOR=2.61; 95%CI:1.68-3.54) and (AOR=4.01; 95% CI: -1.85-9.87) respectively. By the study (26), Neonates with one or more episodes of seizure and asphyxia stage II or III HIE revealed that PNHG had the odds of nearly five times and five times compared to their counterparts at (AOR=4.70; 95% CI:3.78-5.62) and (AOR=5.10; 95%CI: 4.18-6.02) respectively. Other single studies (Kasaye, 2021) showed that neonates whose mothers were with GDM and preeclampsia in the latest pregnancy were in the odds of nearly eight times and two times(AOR=7.79; 95%CI:5.87-9.71) and (AOR=2.44; 95% CI: 1.65-3.23) than their counterparts respectively. On the contrary, the study by (27) revealed that neonates delivered via SVD were 72% less likely to develop PNHG than those delivered via CS at (AOR=0.72; -0.35-1.79).

Discussion

This systematic review and meta-analysis were aimed at assessing the pooled prevalence of PNHG and its associated factors in Ethiopia. The pooled prevalence of PNHG was found to be 19.71(95% CI: 16.85-22.56) which was comparable with studies in Iran Tehran (32), China Shanghai (33) and in the USA (34) at the prevalence of 15.50%, 16.90% and a range of 8-30% respectively.

On the other hand studies from Pakistan used the outcome measurements by RBS cutoff of <50 mg/dl (35), and from [illegible]el by using the RBS cutoffs of <40mg/dl and 47mg/dl (36) were found that the neonatal hypoglycemia was much lower than this review accounting for 0.4% and 3.4-12.10% respectively. This might be because this review utilized the operational definition of persistent neonatal hypoglycemia type and the literature aforementioned have used all type of neonatal hypoglycemia. Moreover, the variation in sample size and socio-demographic variables might contribute to this difference.

From subgroup analysis based on RBS cutoff for diagnosing PNHG, the estimate of pooled prevalence obtained by pooling of the studies by RBS cutoff<47 mg/dl was higher by 21% (95%CI: 16-27) than the studies with RBS cutoff <40 mg/dl 18%(95%CI: 15-22). The possible justification for this might be due to the difference between the RBS cutoff points thereby <47 mg/dl has a higher probability by which more neonates would have RBS measurement in that range. This result is supported by studies in Israel that the prevalence of neonatal hypoglycemia substantially differs between the RBS cutoff <40mg/dl and <47 mg/dl at 3.4% and 12.10% respectively (36). Similarly, there was a difference in the pooled estimate of PNHG between the primary studies with a sample size >400 at 18% (95% CI: 15-22) and those with ≤400 at 21% (95%CI: 17-26). This difference can be explained that studies with sample size variation effected in the outcome of data analysis. Hence, the sample size >400 had relatively more strength to figure out the truth value statistically in the regression analysis than that of ≤400.

This meta-analysis disclosed that preterm birth was 3 times more likely to develop PNHG compared to term neonates. This could be because preterm neonates are uniquely predisposed to hypoglycemia of any type and its related complications due to their limited glycogen and fat stores by which body thermoregulation can be maintained. Premature neonates' inability to generate new glucose using gluconeogenesis pathways affected their glucose utilization in their metabolic process. This in turn, requires higher metabolic demands due to a relatively larger brain than term neonates, for they are unable to respond to a counter-regulatory mechanism to hypoglycemia as compared to term neonates (24,37,38). Similarly, another factor associated with PNHG was neonatal hypothermia increasing the odds of PNHG development by 3 to their counterparts with normothermic neonates. The scientific evidence was congruent to this finding in that neonatal hypothermia predisposes to neonatal hypoglycemia. Neonates have a metabolic response to cooling that involves chemical but nonshivering thermogenesis by sympathetic nerve discharge of norepinephrine in the brown fat. This reaction increases the metabolic rate and oxygen consumption 2-3 fold by activation of glycogen stores, thereby causing transient hypoglycemia as a compensatory mechanism and persistent hypoglycemia as a disorder showing the impaired body glucose hemostasis results in metabolic acidosis and risk of death (39–43).

Neonates who gave birth from mothers with chronic diabetes mellitus were nearly five times more likely to develop PNHG than those from no chronic DM mothers. The justification for this

finding is that infant of a diabetic mother (IDM) is more likely to have a period of low blood sugar level shortly after birth and during the first few days of life. This might be due to the reason that the neonates have been used to getting more sugar than needed from the mother in the uterus before delivery. As this makes the neonates have higher insulin levels than needed after birth, insulin then lowers the blood sugar level by which it takes days for them to adjust to a normal RBS level after birth (44–46).

Regarding breastfeeding initiation time, the neonates who initiated breast milk feeding after an hour had about three times higher Odds (AOR=3.26; 95% CI: 2.03-4.49) to develop PNHG than those breastfed within an hour after delivery (23,24). This evidence with the pooled ORs estimate was highly reflected in the single study by indicating the neonates who delivered via SVD were 72% less likely (AOR=0.72; -0.35-1.79) to develop PNHG compared to CS-delivered neonates (27). This is justified by breast milk is the ideal food for infants that protect against neonatal hypoglycemia. The initiation of breastfeeding within an hour is the mainstay to prevent persistent neonatal hypoglycemia as per WHO recommendations. More importantly, neonates delivered by CS were 2.01 times more likely to develop neonatal hypoglycemia. Hence, any causes that delay breastfeeding within one hour including CS delivery should be intervened. Because, delay breastfed results in neonatal hypoglycemia because breast milk provides all the energy and nutrients that the infant needs for the first months of life and continues to provide up to half or more of a child's nutritional needs (47–50).

On the other hand, neonates with pathological jaundice had about 2.37 higher odds (AOR=2.37; 95%CI: 1.91-2.84) than those who had not(25,27). This finding is comparable with other studies in that neonates with hyperbilirubinemia were 1.81 and 1.93 times more likely to develop neonatal hypoglycemia than those with normal values of the bilirubin test. In another study, neonates treated by phototherapy developed neonatal hypoglycemia at 8.80% (50–52).

This might be since the relationship between neonatal hypoglycemia and neonatal hyperbilirubinemia in pathological jaundice might be mutually influenced in that the OGTT 2-h glucose affected neonatal hypoglycemia. Abnormal postprandial glucose levels suggested impaired glucose tolerance and β cell dysfunction on a physiological basis due to jaundice. As a result, neonates with jaundice would be affected by the physiological process of gluconeogenesis mechanisms to keep the norm-glycemic state.

The findings from a single primary study, being diagnosed by early neonatal sepsis had higher odds of developing neonatal hypoglycemia at (AOR=2.61; 95%CI:1.68-3.54) in this review, this finding was supported by the pieces of literature that early onset of neonatal sepsis (EONS) with neonatal hypoglycemia were about 8.7% and 9.6% of among admitted neonates for sepsis. This is because neonates with neonatal sepsis tend to refuse to breastfeed, resulting in low blood sugar. Similarly, increased metabolic demand is caused by low glucose in sepsis and hypothermia (53,54). By the study (26), Neonates with one or more episodes of seizure and asphyxia stage II or III HIE revealed that PNHG had the odds of nearly five times and five times compared to their counterparts at (AOR=4.70; 95% CI:3.78-5.62) and (AOR=5.10; 95%CI: 4.18-6.02) respectively. This increased risk for hypoglycemia might be due to a lack of storage in the combination of high insulin levels that predisposes them to risk for hypoglycemia. Perinatal asphyxia (PNA) and perinatal seizure secondary to PNA increases the risk of hyperinsulinism in the neonatal period because of the use of anaerobic metabolism to maintain blood glucose concentration. because of the use of anaerobic metabolism to maintain blood glucose concentrations (55). Finally, the study(23) showed that neonates of gestational diabetes mellitus (GDM) and preeclampsia in the latest pregnancy were in the odds of nearly eight times and two times(AOR=7.79; 95%CI:5.87-9.71) and (AOR=2.44; 95% CI: 1.65-3.23) than their counterparts respectively. This can be explained that the most common complication in infants born from GDM mothers is hypoglycemia. This is due to glucose freely passing through the placenta thereby, maternal hyperglycemia associated with GDM results in elevated glucose levels in the fetus causing excess fetal insulin production termed hyperinsulinism (56).

Contribution of the Review

Having the national prevalence estimate and associated factors of persistent neonatal hypoglycemia (PNHG), the policymakers would have evidence-based decisions for neonatal health perspectives in the treatment and prevention of neonatal hypoglycemia in health care settings. Hence, healthcare professionals including nurses, doctors, and others who work in the neonatal intensive care unit (NICU) and the directors of that services can adopt this finding in incorporating it as the priority concern in the neonatal care standards in Ethiopia.

Conclusion and recommendation

Persistent neonatal hypoglycemia was quite common in Ethiopia. The pooled prevalence estimate of PNHG from eight studies was nearly ten percent. Neonates delivered prematurely, neonates with hypothermia, neonates of Diabetic mothers, (IDM), neonates who have been breastfeeding after an hour and neonates with pathologic jaundice had higher odds to develop PNHG than their counterparts. By single primary studies, neonatal sepsis, VLBW, Neonates with one or more episodes of seizure, and asphyxia stage II or III HIE, neonates of GDM mothers had higher odds of developing neonatal hypoglycemia. On the contrary, neonates delivered by SVD were protective against neonatal hypoglycemia. As per this review, the pooled prevalence of PNHG was significant. Several factors associated with were pooled so that the intervention strategies would be required to reduce and prevent the occurrence of it while treating the neonates in the neonatal intensive care units in Ethiopia. Thus, we the team of this review strongly advise health care professionals including nurses, doctors, and others working in NICU to monitor and regulate these and other determining variables that predispose to neonatal hypoglycemia, particularly the persistent type.

Strength and limitation of this study

This review and meta-analysis used a robustly designed systematic review and meta-analysis, using previously tested tools, was used to determine the pooled prevalence and associated factors of persistent neonatal hypoglycemia. Carefully and extensive search strategy used so that more than [illegible]0 neonates were included. This review was the first to bring the pooled prevalence estimate and associated factors in Ethiopia as well as in the globe which provides basis of how big the problem exists and basis for further studies on the effect it predisposes the neurodevelopment of the neonates. As the issue of persistent neonatal hypoglycemia was not well researched, there were limited studies even though larger size of samples used.

Abbreviations

CS: Cesarean section, **GDM**: gestational debates mellitus, **HIE**: hypoxic ischemic encephalopathy, **IDM**: infant of Diabetes mother, **PNHG:** persistent neonatal hypoglycemia

SGA: small for gestational age, **SVD**: spontaneous vaginal delivery, **VLBW**: very low birth weight

Authors' Contribution

SD: initiation of the idea for review and drafted the protocol and supervised the overall process in this review. SZ: reviewed the drafted protocol and writing up edition. AK: data extraction and data analysis. TM: result writing up edition. YT: data extraction team members. AS: revising the result draft writing. WN: the analysis of the extracted data was done by him. KA: reviewed the scientific basis and background about the disorder. DK: edited the final manuscript and revised the result.

Funding

No funding

Ethics approval

As this is a review, not applicable.

Data availability statement

Data are available by reasonable request.

Conflicts of interest

All authors declared that no conflicts of interests

References

1. Cornblath M, Hawdon JM, Williams AF, Aynsley-Green A, Ward-Platt MP, Schwartz R, et al. Controversies regarding definition of neonatal hypoglycemia: Suggested operational thresholds. Pediatrics. 2000;105(5):1141–5.
2. Paul S, Yismaw Fantahun B. Prevalence and Risk factors of Hypoglycaemia in Neonates at: A Cross Sectional Study. 2020;1–15.
3. Thompson-Branch A, Havranek T. Neonatal hypoglycemia. Pediatr Rev. 2017;38(4):147–

57.

4. Abramowski A, Ward R, Hamdan AH. Neonatal hypoglycemia. In: StatPearls [Internet]. StatPearls Publishing; 2021.

5. McKinlay CJD, Alsweiler JM, Ansell JM, Anstice NS, Chase JG, Gamble GD, et al. Neonatal glycemia and neurodevelopmental outcomes at 2 years. N Engl J Med. 2015;373(16):1507–18.

6. Tin W. Defining neonatal hypoglycaemia: a continuing debate. In: Seminars in Fetal and Neonatal Medicine. Elsevier; 2014. p. 27–32.

7. Gustafsson J. Neonatal energy substrate production. Indian J Med Res. 2009;130(5):618–23.

8. Frank-Briggs AI, Ojule AC, Nkanginieme KE. Neonatal hypoglycaemia: prevalence and clinical manifestations in Port Harcourt, Nigeria. Port Harcourt Med J. 2008;2(2):166–70.

9. Shah R, Harding J, Brown J, McKinlay C. Neonatal glycaemia and neurodevelopmental outcomes: a systematic review and meta-analysis. Neonatology. 2019;115(2):116–26.

10. Sultan OM. The Hidden Burden of Hypoglycemia in Neonatal Mortality in Tanzania-The Case of Saint Francis Hospital, Ifakara. IMTU Med J. 2012;3:34–9.

11. Kush ML, Baschat AA. Fetal responses to placental insufficiency. In: Textbook of Perinatal Medicine. CRC Press; 2006. p. 1021–38.

12. Menni F, De Lonlay P, Sevin C, Touati G, Peigné C, Barbier V, et al. Neurologic outcomes of 90 neonates and infants with persistent hyperinsulinemic hypoglycemia. Pediatrics. 2001;107(3):476–9.

13. Tam EWY, Widjaja E, Blaser SI, MacGregor DL, Satodia P, Moore AM. Occipital lobe injury and cortical visual outcomes after neonatal hypoglycemia. Pediatrics. 2008;122(3):507–12.

14. McKinlay CJD, Alsweiler JM, Anstice NS, Burakevych N, Chakraborty A, Chase JG, et al. Association of neonatal glycemia with neurodevelopmental outcomes at 4.5 years. JAMA Pediatr. 2017;171(10):972–83.

15. You D, Hug L, Ejdemyr S, Idele P, Hogan D, Mathers C, et al. Global, regional, and national levels and trends in under-5 mortality between 1990 and 2015, with scenario-based projections to 2030: a systematic analysis by the UN Inter-agency Group for Child Mortality Estimation. Lancet. 2015;386(10010):2275–86.

16. McGowan JE. Neonatal hypoglycemia. Pediatr Rev. 1999;20(7):e6–15.

17. Gebremichael SG, Fenta SM. Under-Five Mortality and Associated Risk Factors in Rural Settings of Ethiopia: Evidences from 2016 Ethiopian Demographic and Health Survey. Adv Public Heal. 2020;2020.

18. Page MJ, McKenzie JE, Bossuyt PM, Boutron I, Hoffmann TC, Mulrow CD, et al. The

PRISMA 2020 statement: An updated guideline for reporting systematic reviews. BMJ. 2021;372.

19. Peters MDJ, Godfrey CM, McInerney P, Soares CB, Khalil H, Parker D. The Joanna Briggs Institute reviewers' manual 2015: methodology for JBI scoping reviews. 2015;

20. Rothstein HR, Sutton AJ, Borenstein M. Publication bias in meta- analysis. Publ bias meta- analysis Prev Assess Adjust. 2005;1–7.

21. Ioannidis JPA. Interpretation of tests of heterogeneity and bias in meta- analysis. J Eval Clin Pract. 2008;14(5):951–7.

22. Borenstein M, Hedges L V, Higgins JPT, Rothstein HR. A basic introduction to fixed- effect and random- effects models for meta- analysis. Res Synth Methods. 2010;1(2):97–111.

23. Kasaye F. College Of Health Science School Of Nursing And Midwifery Department Of Nursing Prevalence And Predictors Of Hypoglycemia Among Neonates On Admission To Neonatal Intensive Care Unit In Selected Public Hospitals , Principal Investigator : Fikirte Kasaye (. 2021;

24. Sertsu A, Nigussie K, Eyeberu A, Tibebu A, Negash A, Getachew T, et al. Determinants of neonatal hypoglycemia among neonates admitted at Hiwot Fana Comprehensive Specialized University Hospital, Eastern Ethiopia: A retrospective cross-sectional study. SAGE Open Med. 2022;10.

25. Yohannes M. Magnitude Of Jaundice And Associated Factors Among Neonates Admitted To Neonatal In_Tensive Care Unit In Eastern Ethiopia. Front Neurosci. 2021;14(1):1–13.

26. Chanie ES, Shiferaw SM, Feleke DG, Bantie B, Moges N, Tasew SF, et al. Maximizing the detection rate of hypoglycemia among preterm neonates admitted in Neonatal intensive care unit in Ethiopia, 2021. Sci Rep [Internet]. 2023;13(1):1–7. Available from: https://doi.org/10.1038/s41598-023-29112-y

27. Demis S, Munye T, Agmas K, Necho W, Moges N. Incidence of Persistent Neonatal Hypoglycemia and Associated Factors among Neonatal Intensive Care Unit Admissions in South Gondar Public Hospitals , Northcentral Ethiopia : a prospective cross sectional study. unpublished. 2022;1–24.

28. Demisse AG, Alemu F, Gizaw MA, Tigabu Z. Patterns of admission and factors associated with neonatal mortality among neonates admitted to the neonatal intensive care unit of University of Gondar Hospital, Northwest Ethiopia. Pediatr Heal Med Ther. 2017;Volume 8:57–64.

29. Bogale MT, Bogale WA, Kassie DG, Woldesellassie A, Tamiru AT. Factors associated with neonatal hyperbilirubinemia in case files of all admitted inborn and outborn neonates in Northwest Ethiopia in 2019. Iran J Neonatol. 2021;12(1):46–53.

30. Fantahun B, Nurussen I. Prevalence and risk factors of neonatal hypoglycaemia at st. Paul's Hospital Millennium Medical College, Ethiopia. Pediatr Child Heal [Internet].

2021;26(1):13. Available From: Https://Www.Researchgate.Net/Profile/Bereket-Fantahun 2/Publication/358834078_ORIGINAL_ARTICLE_PREVALENCE_AND_RISK_FACTORS_OF_NEONATAL_HYPOGLYCAEMIA_AT_ST_PAUL'S_HOSPITAL_MILLENNIUM_MEDICAL_COLLEGE_ETHIOPIA/Links/6217e57e6738db292ac385ac/ORIGINAL-ARTICLE

31. Briggs J. Checklist for Systematic Reviews and Research Syntheses [Internet]. The Joanna Briggs Institute. 2017. p. 7. Available from: http://joannabriggs.org/research/critical-appraisal-tools.htmlwww.joannabriggs.org%0Awww.joannabriggs.org

32. Dashti N, Einollahi N, Abbasi S. Neonatal Hypoglycemia : Prevalence And Clinical Manifestations In Tehran Children ' S Hospital. 2007;23(3).

33. Zhou W, Yu J, Wu Y, Zhang H. Hypoglycemia incidence and risk factors assessment in hospitalized neonates neonates. 2015;7058:6–10.

34. Alemu BT, Olayinka O, Baydoun HA, Hoch M, Elci MA. Neonatal Hypoglycemia in Diabetic Mothers : a Systematic Review. 2017;21:42–53.

35. Najati N, Saboktakin L. Prevalence and underlying etiologies of neonatal hypoglycemia. Vol. 13, Pakistan Journal of Biological Sciences. 2010. p. 753–6.

36. Bromiker R, Perry A, Kasirer Y, Einav S, Klinger G. Early neonatal hypoglycemia : incidence of and risk factors . A cohort study using universal point of care screening. J Matern Neonatal Med [Internet]. 2019;32(5):786–92. Available from: https://doi.org/10.1080/14767058.2017.1391781

37. Sharma A, Davis A, Shekhawat PS. Hypoglycemia in the preterm neonate: etiopathogenesis, diagnosis, management and long-term outcomes. Transl Pediatr. 2017 Oct;6(4):335–48.

38. Goode RH, Rettiganti M, Li J, Lyle RE, Whiteside-Mansell L, Barrett KW, et al. Developmental outcomes of preterm infants with neonatal hypoglycemia. Pediatrics. 2016;138(6).

39. Agardh CD, Smith ML, Siesjö BK. The influence of hypothermia on hypoglycemia-induced brain damage in the rat. Acta Neuropathol. 1992;83(4):379–85.

40. Kedes LH, Field JB. Hypothermia. N Engl J Med [Internet]. 1964 Oct 8;271(15):785–7. Available from: https://doi.org/10.1056/NEJM196410082711509

41. Strauch, Barry S.Felig, PhilipBaxter JD. 2023. 2023.

42. Yitayew YA, Aitaye EB, Lechissa HW, Gebeyehu LO. Neonatal Hypothermia and Associated Factors among Newborns Admitted in the Neonatal Intensive Care Unit of Dessie Referral Hospital, Amhara Region, Northeast Ethiopia. Int J Pediatr. 2020;2020:3013427.

43. Tran C, Gariani K, Herrmann FR, Juan L, Philippe J, Rutschmann OT, et al. Hypothermia is a frequent sign of severe hypoglycaemia in patients with diabetes. Diabetes Metab.

2012 Oct;38(4):370–2.

44. Kallem VR, Pandita A, Pillai A. Infant of diabetic mother: what one needs to know? J Matern Neonatal Med [Internet]. 2020;33(3):482–92. Available from: https://doi.org/10.1080/14767058.2018.1494710

45. Mormile R. Neonates of diabetic mothers: The starting point for developing novel therapeutic approaches to ischemic heart and brain? Med Hypotheses. 2016 Nov;96:75–7.

46. Opara PI, Jaja T, Onubogu UC. Morbidity and mortality amongst infants of diabetic mothers admitted into a special care baby unit in Port Harcourt, Nigeria. Ital J Pediatr. 2010;36(1):2–7.

47. Ekubay M, Berhe A, Yisma E. Initiation of breastfeeding within one hour of birth among mothers with infants younger than or equal to 6 months of age attending public health institutions in Addis Ababa, Ethiopia. Int Breastfeed J. 2018;13(1):1–7.

48. WHO. Guideline:Protecting, promoting and supporting breastfeeding in facilities providing maternity and newborn services [Internet]. World Health OrganizationWHO. 2017. 1–136 p. Available from: https://apps.who.int/iris/bitstream/handle/10665/259386/9789241550086-eng.pdf

49. Karim F, Masum Billah SK, Chowdhury MAK, Zaka N, Manu A, Arifeen S El, et al. Initiation of breastfeeding within one hour of birth and its determinants among normal vaginal deliveries at primary and secondary health facilities in Bangladesh: A case-observation study. PLoS One. 2018;13(8):1–15.

50. He J, Song J, Zou Z, Fan X, Tian R, Xu J, et al. Association between neonatal hyperbilirubinemia and hypoglycemia in Chinese women with diabetes in pregnancy and influence factors. Sci Rep [Internet]. 2022;12(1):16975. Available from: https://doi.org/10.1038/s41598-022-21114-6

51. Kallem VR, Pandita A, Pillai A. Infant of diabetic mother: what one needs to know? J Matern neonatal Med Off J Eur Assoc Perinat Med Fed Asia Ocean Perinat Soc Int Soc Perinat Obstet. 2020 Feb;33(3):482–92.

52. Salman M, Rathore H, Arif S, Ali R, Khan AA, Nasir M. Frequency of Immediate Neonatal Complications (Hypoglycemia and Neonatal Jaundice) in Late Preterm and Term Neonates. Cureus. 2021 Jan;13(1):e12512.

53. Islam MS, Mia MAH, Akhter KR, Haque M, Malik M. Glycemic Status and its Effect in Neonatal Sepsis in a Tertiary Care Hospital. Bangladesh J Child Heal. 2017;40(1):21–5.

54. Ellahony D, El-Mekkawy M, A. Issa AHR. Prevalence of glucose level abnormalities in neonatal sepsis and its association with mortality. Menoufia Med J. 2020;33(1):127.

55. Sweet CB, Grayson S, Polak M. Management strategies for neonatal hypoglycemia. J Pediatr Pharmacol Ther JPPT Off J PPAG. 2013 Jul;18(3):199–208.

56. Voormolen DN, de Wit L, van Rijn BB, DeVries JH, Heringa MP, Franx A, et al.

Neonatal Hypoglycemia Following Diet-Controlled and Insulin-Treated Gestational Diabetes Mellitus. Diabetes Care [Internet]. 2018 Apr 13;41(7):1385–90. Available from: https://doi.org/10.2337/dc18-0048

Figure 1

Click here to access/download;Figure;fig. 1.docx

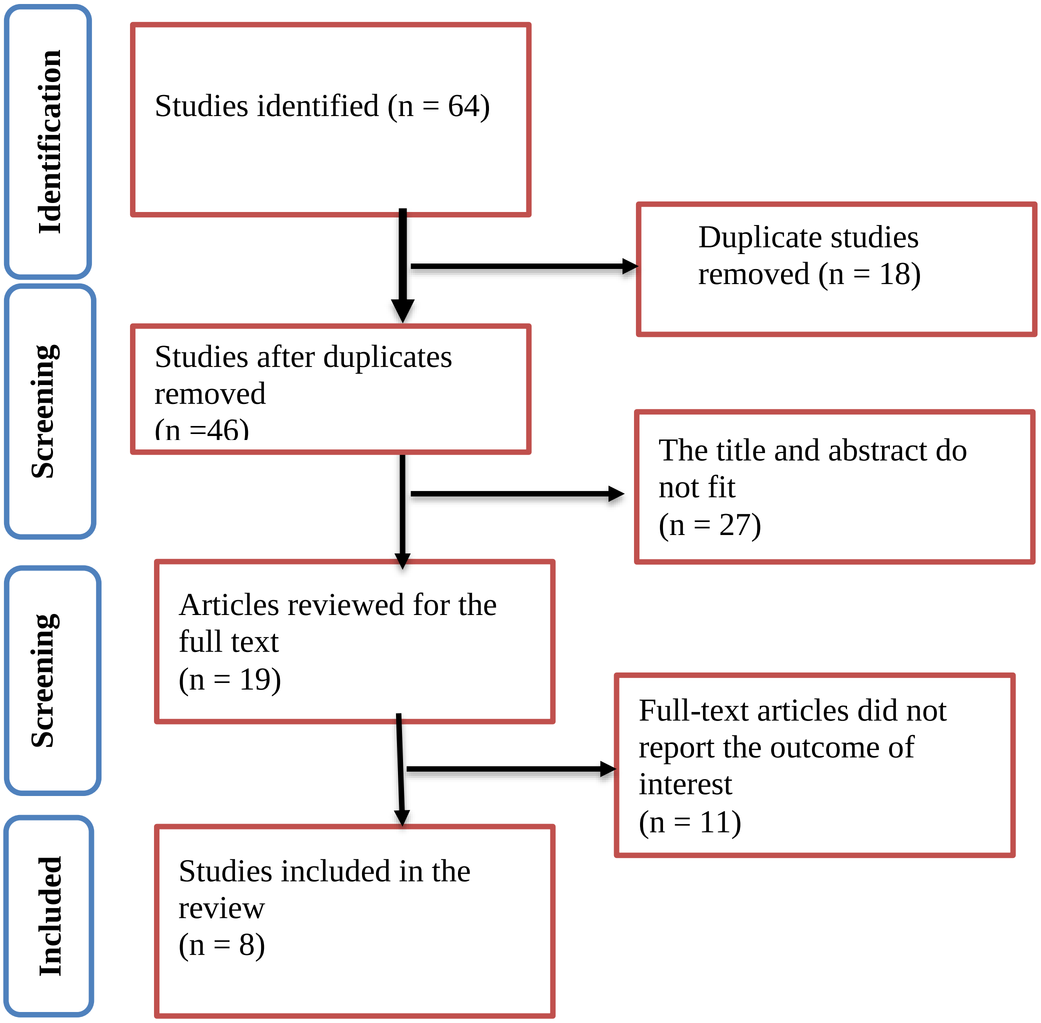

Figure 1. Study selection process

Figure 2 Click here to access/download;Figure;Fig. 2 Forest plot.docx

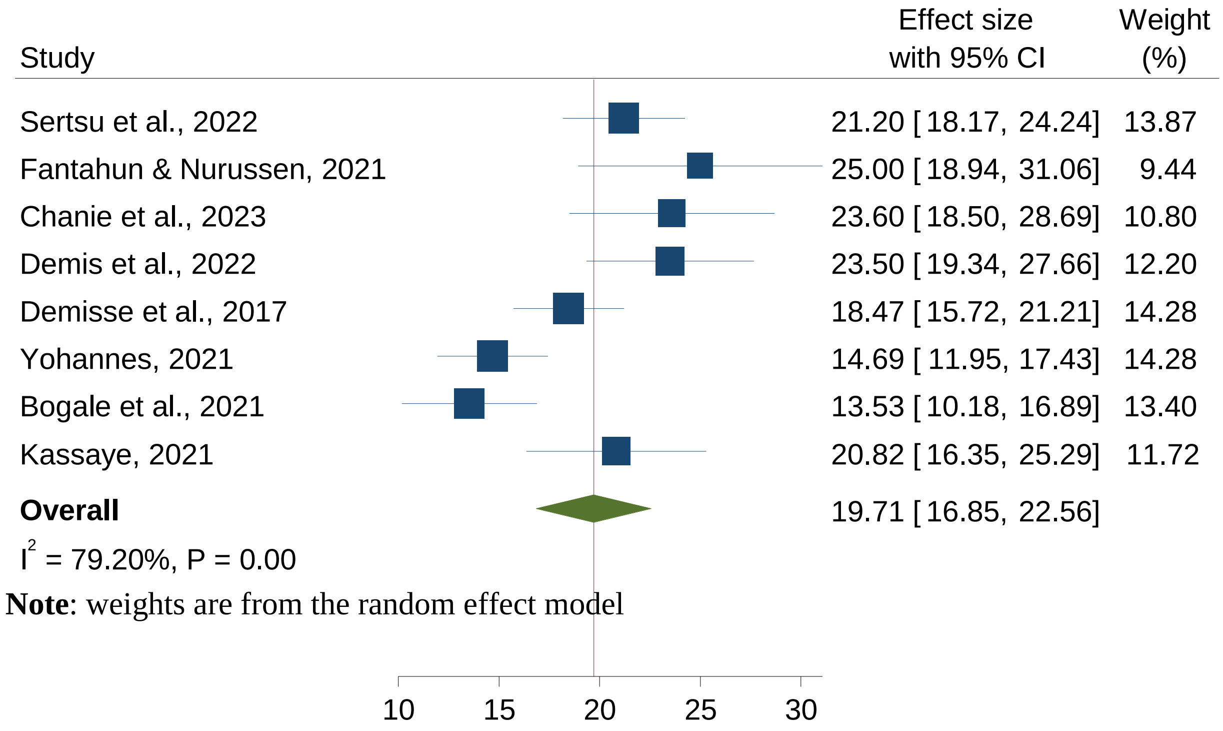

Figure 2. Forest plot for the pooled prevalence of PNHG in Ethiopia, 2023.

Figure 3

Click here to access/download;Figure;fig. 3.docx

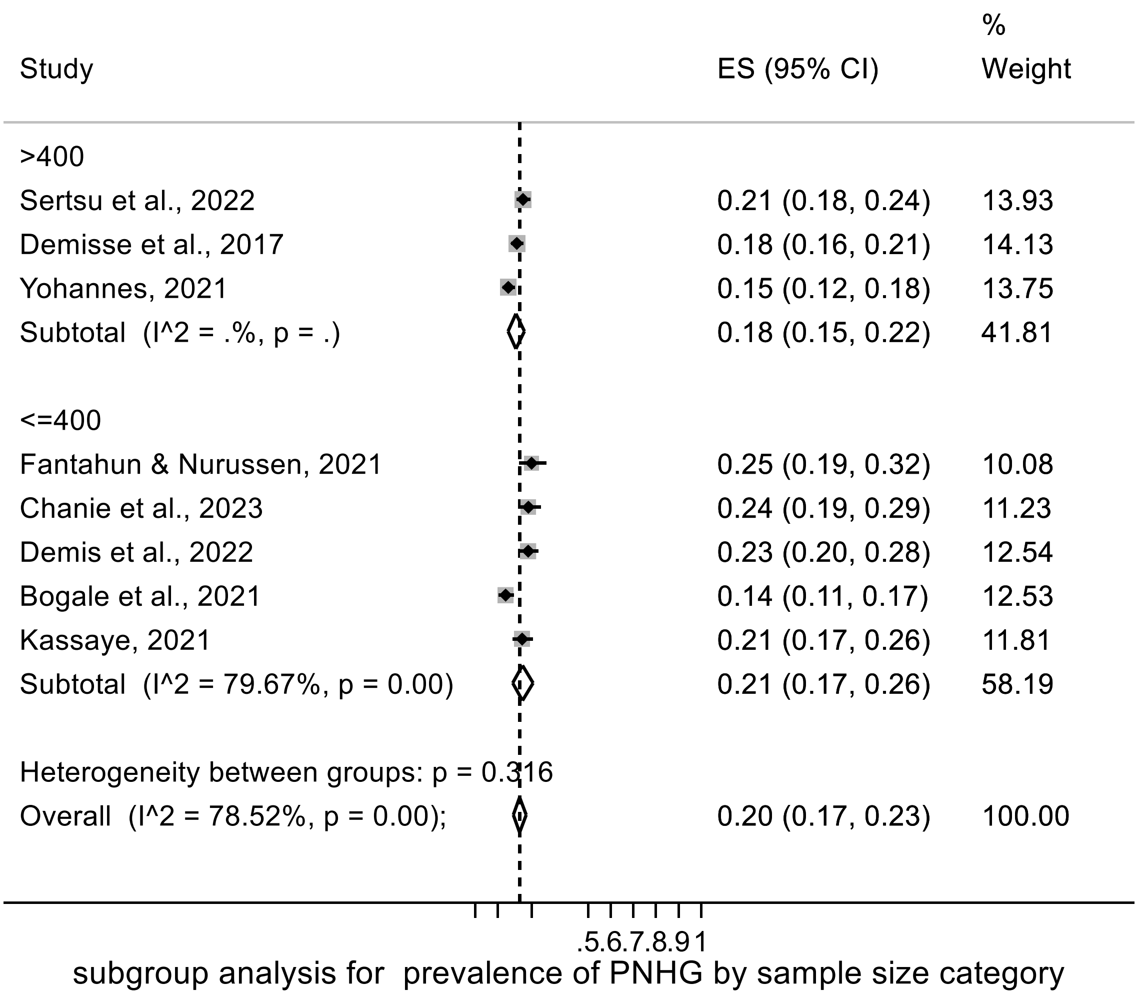

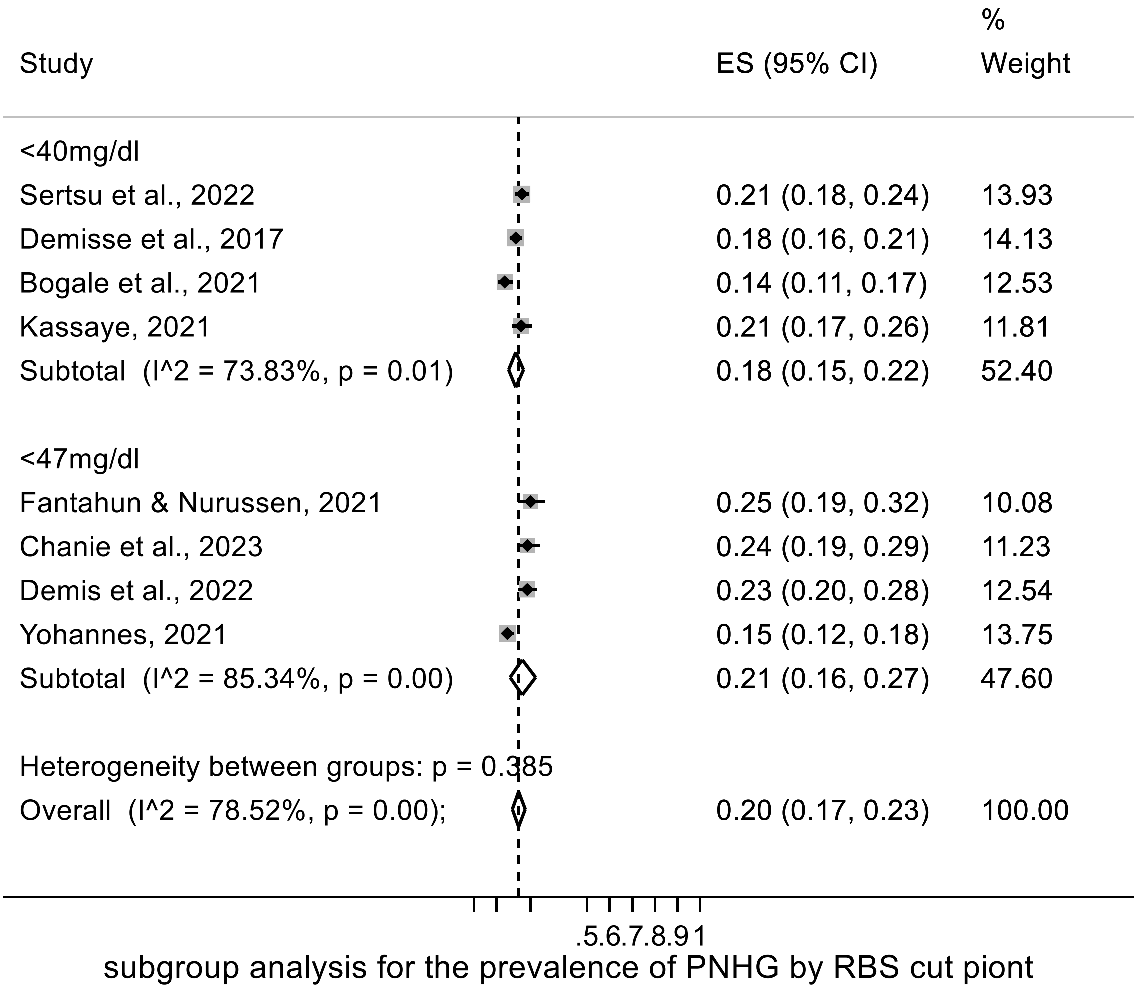
Study
ES (95% CI)
% Weight
<40mg/dl
Sertsu et al., 2022 0.21 (0.18, 0.24) 13.93
Demisse et al., 2017 0.18 (0.16, 0.21) 14.13
Bogale et al., 2021 0.14 (0.11, 0.17) 12.53
Kassaye, 2021 0.21 (0.17, 0.26) 11.81
Subtotal (I^2 = 73.83%, p = 0.01) 0.18 (0.15, 0.22) 52.40
<47mg/dl
Fantahun & Nurussen, 2021 0.25 (0.19, 0.32) 10.08
Chanie et al., 2023 0.24 (0.19, 0.29) 11.23
Demis et al., 2022 0.23 (0.20, 0.28) 12.54
Yohannes, 2021 0.15 (0.12, 0.18) 13.75
Subtotal (I^2 = 85.34%, p = 0.00) 0.21 (0.16, 0.27) 47.60
Heterogeneity between groups: p = 0.385
Overall (I^2 = 78.52%, p = 0.00); 0.20 (0.17, 0.23) 100.00
.5.6.7.8.91
subgroup analysis for the prevalence of PNHG by RBS cut piont

Supplementary file 1

Click here to access/download
Supporting Information
PRISMA_2020_checklist (3).docx

Supplementary file 2

Click here to access/download
Supporting Information
Quality Checklist PNHG sup. 2.docx
