# Supplementary material for: Prevalence of prolonged transitional neonatal hypoglycemia and associated factors in Ethiopia: A systematic review and meta-analysis
Source: PLoS One. 2025 Feb 6;20(2):e0316464. doi: 10.1371/journal.pone.0316464 (PMC11801580; doi:10.1371/journal.pone.0316464)
Supplement: Attachment — Submitted filename: PONE-D-23-19848_R1_reviewer.pdf [file pone.0316464.s008.pdf]

# PLOS ONE

# Prevalence of prolonged transitional neonatal hypoglycemia and associated factors in Ethiopia: A systematic review and meta-analysis

--Manuscript Draft--

| | |
|---|---|
| **Manuscript Number:** | PONE-D-23-19848R1 |
| **Article Type:** | Research Article |
| **Full Title:** | Prevalence of prolonged transitional neonatal hypoglycemia and associated factors in Ethiopia: A systematic review and meta-analysis |
| **Short Title:** | Prevalence of prolonged transitional neonatal hypoglycemia and its associated factors in Ethiopia |
| **Corresponding Author:** | Solomon Demis<br>Debre Tabor University<br>Debre Tabor, South Gondar ETHIOPIA |
| **Keywords:** | Neonatal hypoglycemia, prolonged transitional hypoglycemia, abnormal neonatal hypoglycemia, incidence of hypoglycemia, newborn blood glucose level, hyperinsulinism |
| **Abstract:** | Introduction: Most neonates have transient hypoglycemia, which responds to treatment and is associated with a good prognosis compared with prolonged hypoglycemia. Hypoglycemia occurring after 48 hours of age is termed prolonged transitional hypoglycemia and results in abrupt injury and neurodevelopmental defects. Method: A weighted inverse-variance random effects model was used. Variations across the studies were checked using a forest plot, I2 statistics, and Egger's test. Data was extracted from 20-27 May 2023 for studies since 2020. A random blood sugar (RBS) concentration <47 mg/dL at 48-72 hours after birth was used to indicate prolonged transitional neonatal hypoglycemia (PTNHG). Results: Eight studies with a sample of 3686 neonates were included. The pooled prevalence of prolonged PTNHG was 19.71% (95% CI: 16.85-22.56); I2=79.20, P<0.001). By subgroup analysis from sample sizes > 400 and ≤ 400, PTNHG was found to be comparable at 18% (15-22) and 21% (17-26), respectively. On the other hand, when using RBS cutoffs <47 mg/dL and <40 mg/dL, the PTNHGs were relatively similar at 21% (16-27) and 18% (15-22), respectively. Preterm birth (AOR=3.31; 95% CI=2.57-4.04), hypothermia (AOR=3.41; 95% CI=2.19-4.62), infant of diabetes mother (IDM) (AOR=4.71; 95% CI=2.15-7.26), breastfeeding initiation after an hour (AOR=3.26; 95% CI=2.03-4.49), and pathological jaundice (AOR=2.37; 95% CI=1.91-2.84) were significantly associated with PTNHG. Conclusion: Nearly one-fifth of the neonates were in a state of hypoglycemia during their hospitalization. Fortunately, the factors were easily modifiable. Prioritization of the problem in current national NICU hospital practice guidelines, initiation of early breastfeeding, particularly for cesarean section (CS) delivery and IDM would reduce the burden. |
| **Order of Authors:** | Solomon Demis Kebede |
| | Amare kassaw |
| | Tigabu Munye Aytenew |
| | Kindu Agmas |
| | Demewoz Kefale |
| **Response to Reviewers:** | Authors’ response to reviewers’ comments<br>“Prevalence of persistent neonatal hypoglycemia and its associated factors in Ethiopia: A systematic review and meta-analysis”<br>Good beginning; yet few areas of improvement and the readers/reviewers concerns / suggestion to be incorporated<br>1.Good title to capture the reader of different healthcare professionals<br>2.In the abstract it is mentioned; the sample size was 3686 from the 8 studies but later on it is mentioned 3600? It needs to be clear for the readers<br>Authors’ response: revised<br>3.Interested background and introduction; keeps the readers indulged in the reading; |

Powered by Editorial Manager® and ProduXion Manager® from Aries Systems Corporation

| | |
|---|---|
| | very good<br>4.Good rationale for the study and gives the readers novelty<br>5.PICO format has the biggest concerns: Readers are totally confused at once; sudden change from the hypoglycemia to intra-Ventricular hemorrhage???? Does that align with the title of the study? Turning point from the winners to losers in the eyes of the readers? Unbearable as reader is not happy with the twist; clear the reader what the study is about?<br>Authors' response: revised and we ask for pardon. We made writing errors.<br>6.Completely opposing from the title as per readers best judgment??? Derailed from the track? I don't know how and why it happened??? Many questions arise. As stated in PICO format, (the following review questions were developed for the search for data. What is the magnitude of IVH in African countries? What are the predictors of IVH in African countries?) Reader is confused, what the outcome is stated and what it should be as per the title of the study???<br>Authors' response: yes we did it correct.<br>7.The key terms in the abstract are mentioned as "Prevalence, persistent neonatal hypoglycemia, prolonged neonatal hypoglycemia, Ethiopia (these were the key words as stated earlier in the abstract); suddenly all these are mentioned later on as "The core search terms and phrases were "prevalence", "incidence", "epidemiology", "proportion", "magnitude", "burden", "Intraventricular" and ''predictors'', "risk factors'', ''associated factors'' and ''Africa'' Or ''African countries"???? Huge gap exists<br>Authors' response: corrected typos errors<br>8.time frame in the inclusion criteria need to be mentioned<br>Authors' response: the articles included were those published from 2020 to 2022.<br>9.Pakistan; Iran and Israel are from different region (apart from Ethiopia); do these studies align with the inclusion criteria??? Referring to the second Para of the discussion chapter?<br>Authors' response: this is to insight the burden across the globe…not to analyze It is aimed to show the difference in different regions but not part for this review.<br>10.Referring to Critical appraisal and quality assessment portion ; it would be more appropriate if the examples be given how the criteria was accomplished in the particular study?<br>Authors' response: yes the comment is accepted and given full reference to supplementary file 2.<br>11.Strength and limitation of this study depicts 3600 neonates were included??? Does that mean a sample size??? If yes, previously it was mentioned that 3686 neonates were the ample size? Clarity is suggested<br>Authors' response: revised and sorry for repeated writing errors.<br>12.Data extraction date and authors' put beneath the table and that was from 20-27 May 2023.<br>13.The article by Demis et al., 2022 was accepted for publication and that is why we cited as it was there.<br>FUNDING INFORMATION:<br>14. No specific funding was received for this work.<br>DATA AVAILABILITY<br>15. Data was uploaded as supporting information. |
| **Additional Information:** | |
| **Question** | **Response** |
| **Financial Disclosure**<br><br>Enter a financial disclosure statement that describes the sources of funding for the work included in this submission. Review the submission guidelines for detailed requirements. View published research articles from *PLOS ONE* for specific examples.<br><br>This statement is required for submission and **will appear in the published article** if | No |

| | |
|---|---|
| the submission is accepted. Please make sure it is accurate.<br><br>**Unfunded studies**<br>Enter: *The author(s) received no specific funding for this work.*<br><br>**Funded studies**<br>Enter a statement with the following details:<br>• Initials of the authors who received each award<br>• Grant numbers awarded to each author<br>• The full name of each funder<br>• URL of each funder website<br>• Did the sponsors or funders play any role in the study design, data collection and analysis, decision to publish, or preparation of the manuscript?<br>• **NO** - Include this sentence at the end of your statement: *The funders had no role in study design, data collection and analysis, decision to publish, or preparation of the manuscript.*<br>• **YES** - Specify the role(s) played.<br><br>* typeset | |
| **Competing Interests**<br><br>Use the instructions below to enter a competing interest statement for this submission. On behalf of all authors, disclose any competing interests that could be perceived to bias this work—acknowledging all financial support and any other relevant financial or non-financial competing interests.<br><br>This statement is required for submission and **will appear in the published article** if the submission is accepted. Please make sure it is accurate and that any funding sources listed in your Funding Information later in the submission form are also declared in your Financial Disclosure statement.<br><br>View published research articles from *PLOS ONE* for specific examples. | No authors have competing interests. |

| | |
|---|---|
| **NO authors have competing interests**<br><br>Enter: *The authors have declared that no competing interests exist.*<br><br>**Authors with competing interests**<br><br>Enter competing interest details beginning with this statement:<br><br>*I have read the journal's policy and the authors of this manuscript have the following competing interests: [insert competing interests here]*<br><br>* typeset | |
| **Ethics Statement**<br><br>Enter an ethics statement for this submission. This statement is required if the study involved:<br><br>• Human participants<br>• Human specimens or tissue<br>• Vertebrate animals or cephalopods<br>• Vertebrate embryos or tissues<br>• Field research<br><br>Write "N/A" if the submission does not require an ethics statement.<br><br>General guidance is provided below. Consult the submission guidelines for detailed instructions. **Make sure that all information entered here is included in the Methods section of the manuscript.** | Not applicable. |

| | |
|---|---|
| **Format for specific study types**<br><br>**Human Subject Research (involving human participants and/or tissue)**<br>• Give the name of the institutional review board or ethics committee that approved the study<br>• Include the approval number and/or a statement indicating approval of this research<br>• Indicate the form of consent obtained (written/oral) or the reason that consent was not obtained (e.g. the data were analyzed anonymously)<br><br>**Animal Research (involving vertebrate animals, embryos or tissues)**<br>• Provide the name of the Institutional Animal Care and Use Committee (IACUC) or other relevant ethics board that reviewed the study protocol, and indicate whether they approved this research or granted a formal waiver of ethical approval<br>• Include an approval number if one was obtained<br>• If the study involved *non-human primates*, add *additional details* about animal welfare and steps taken to ameliorate suffering<br>• If anesthesia, euthanasia, or any kind of animal sacrifice is part of the study, include briefly which substances and/or methods were applied<br><br>**Field Research**<br><br>Include the following details if this study involves the collection of plant, animal, or other materials from a natural setting:<br>• Field permit number<br>• Name of the institution or relevant body that granted permission | |
| **Data Availability**<br><br>Authors are required to make all data underlying the findings described fully available, without restriction, and from the time of publication. PLOS allows rare exceptions to address legal and ethical concerns. See the PLOS Data Policy and FAQ for detailed information. | Yes - all data are fully available without restriction |

| | |
|---|---|
| A Data Availability Statement describing where the data can be found is required at submission. Your answers to this question constitute the Data Availability Statement and **will be published in the article**, if accepted.<br><br>**Important:** Stating 'data available on request from the author' is not sufficient. If your data are only available upon request, select 'No' for the first question and explain your exceptional situation in the text box.<br><br>Do the authors confirm that all data underlying the findings described in their manuscript are fully available without restriction? | |
| **Describe where the data may be found in full sentences. If you are copying our sample text, replace any instances of XXX with the appropriate details.**<br><br>• If the data are **held or will be held in a public repository**, include URLs, accession numbers or DOIs. If this information will only be available after acceptance, indicate this by ticking the box below. For example: *All XXX files are available from the XXX database (accession number(s) XXX, XXX.*).<br>• If the data are all contained **within the manuscript and/or Supporting Information files**, enter the following: *All relevant data are within the manuscript and its Supporting Information files.*<br>• If neither of these applies but you are able to provide **details of access elsewhere**, with or without limitations, please do so. For example:<br><br>*Data cannot be shared publicly because of [XXX]. Data are available from the XXX Institutional Data Access / Ethics Committee (contact via XXX) for researchers who meet the criteria for access to confidential data.*<br><br>*The data underlying the results presented in the study are available from (include the name of the third party* | The data will be available upon reasonable request. |

| | |
|---|---|
| *and contact information or URL).*<br>• This text is appropriate if the data are owned by a third party and authors do not have permission to share the data.<br><br>* typeset | |
| Additional data availability information: | Tick here if your circumstances are not covered by the questions above and you need the journal’s help to make your data available. |

*Powered by Editorial Manager® and ProduXion Manager® from Aries Systems Corporation*

Manuscript Click here to access/download;Manuscript;ManuscriptR1.DOCX

# Prevalence of prolonged transitional neonatal hypoglycemia and associated factors in Ethiopia: A systematic review and meta-analysis

**Solomon Demis Kebede[1], Amare Kassaw[1], Tigabu Munye Aytenew[2], Kindu Agmas[3], Demewoz Kefale[1]**

[1] Department of Pediatrics and Child Health Nursing, College of Health Sciences, Debre Tabor University, Debre Tabor, Ethiopia

[2] Department of Nursing, College of Health Sciences, Debre Tabor University, Debre Tabor, Ethiopia

[3] Departments of Pediatrics and Child Health, Debre Tabor Comprehensive Specialized Hospital, Debre Tabor, Ethiopia

***Correspondence**: solomondemis@gmail.com, mob. +251930713008

# Abstract

**Introduction:** Most neonates have transient hypoglycemia, which responds to treatment and is associated with a good prognosis compared with prolonged hypoglycemia. Hypoglycemia occurring after 48 hours of age is termed prolonged transitional hypoglycemia and results in abrupt injury and neurodevelopmental defects. **Method:** A weighted inverse-variance random effects model was used. Variations across the studies were checked using a forest plot, $I^2$ statistics, and Egger's test. Data was extracted from 20-27 May 2023 for studies since 2020. A random blood sugar (RBS) concentration <47 mg/dL at 48-72 hours after birth was used to indicate prolonged transitional neonatal hypoglycemia (PTNHG). **Results**: Eight studies with a sample of 3686 neonates were included. The pooled prevalence of prolonged PTNHG was 19.71% (95% CI: 16.85-22.56); I2=79.20, P<0.001). By subgroup analysis from sample sizes > 400 and ≤ 400, PTNHG was found to be comparable at 18% (15-22) and 21% (17-26), respectively. On the other hand, when using RBS cutoffs <47 mg/dL and <40 mg/dL, the PTNHGs were relatively similar at 21% (16-27) and 18% (15-22), respectively. Preterm birth (AOR=3.31; 95% CI=2.57-4.04), hypothermia (AOR=3.41; 95% CI=2.19-4.62), infant of diabetes mother (IDM) (AOR=4.71; 95% CI=2.15-7.26), breastfeeding initiation after an hour (AOR=3.26; 95% CI=2.03-4.49), and pathological jaundice (AOR=2.37; 95% CI=1.91-2.84) were significantly associated with PTNHG. **Conclusion:** Nearly one-fifth of the neonates were in a state of hypoglycemia during their hospitalization. Fortunately, the factors were easily modifiable. Prioritization of the problem in current national NICU hospital practice guidelines, initiation of early breastfeeding, particularly for cesarean section (CS) delivery and IDM would reduce the burden.

**Prospero ID**: CRD42023424953.

**Keywords**: Neonatal hypoglycemia, prolonged transitional hypoglycemia, abnormal neonatal hypoglycemia, incidence of hypoglycemia, newborn blood glucose level, hyperinsulinism

# Introduction

Neonatal hypoglycemia is defined as low blood glucose concentration below 40-50 mg/dL as per World Health Organization (WHO) and American Academy of Pediatrics (AAP)[1,2]. There are two types of neonatal hypoglycemia: transient and prolonged. Most neonates have transient hypoglycemia, which responds to treatment and is associated with a good prognosis compared with persistent hypoglycemia. Hypoglycemia that occurs after 48 hours of age is termed prolonged transitional hypoglycemia, the clinical manifestations of which are generated in the short term, with abrupt injury and neurodevelopmental sequelae occurring over the long-term [3,4].

Observations have shown that healthy infants experience transient hypoglycemia as a part of the normal adaption to extrauterine life, with a decline in blood glucose concentrations to values as low as 20 to 25 mg/dL in the first two hours of life[3]. Neonatal hypoglycemia persisting beyond 48 hours after birth might be prolonged transitional hypoglycemia[4].

Deviations from the normal transition can occur as a result of a wide range of factors predisposing individuals to neonatal hypoglycemia. The brain uses glucose primarily to meet its metabolic requirements. Therefore, neonatal hypoglycemia is a significant medical emergency in neonatal units. The signs and symptoms are often nonspecific. Some neonates are completely asymptomatic. Many cases, therefore, remain undiagnosed. The diagnosis of neonatal hypoglycemia requires a high index of suspicion since its presentation is different. Early diagnosis and treatment could prevent abrupt signs and symptoms, long-term complications, and death [2,5,6].

The neonatal hypoglycemia is still a severe concern because it causes neurological defect, an increased risk of mortality, and other complications in neonates and families. A study conducted by Shah R et al., (2019) revealed that the risk of a specific cognitive defect increased by 2-3-fold in early childhood. Neonatal hypoglycemia had odds of 3.2 for neurodevelopmental impairment and 2.04 for low literacy at school in late childhood [7]. And, neonates with hypoglycemia would developed cerebral palsy at 8% in their childhood[8].

Moreover, a study in Tanzania showed that hypoglycemia was a major cause of neonatal mortality, contributing to 20% of all causes. Other family-related burdens secondary to neonatal

hypoglycemia admission include increased costs to the family and separation from the mother[9]. Thus, detection and treatment of these groups of neonates are targeted to reduce neonatal death, mainly in low-resource countries [10–13]. According to some studies, neonatal hypoglycemia affects up to approximately 4-5 per 1000 term infants [5,6]. A blood glucose level (RBS) below 47 mg/dL (2.6 mmol/L), if present during the 5 days during the first two months of life, was significantly correlated with acute seizure activity, abnormal neuromotor manifestations, and impaired intellectual performance at 18 months of age[14].

Despite progress over the past two decades, the age distribution of the mortality of children and young adolescents shows that the highest risk of death occurs during the neonatal period (the first 28 days of life), during which preventable causes accounted for 2.5 million out of 6.2 million deaths of children and young adolescents in 2018 alone[3,15]. The neonatal mortality rate was estimated to be 18 deaths/1,000 live births globally[3,15]. The probability of dying after the first month and before reaching age 1 was 11 per 1,000 live births [3,15].

According to the Ethiopian Demographic Health Survey (EDHS), Ethiopia has one of the highest neonatal mortality rates in the world (29 per 1000 live births), which represents poor progress in reducing the rate of neonatal mortality from 2000 to 2016[16]. The report of previous studies on the prevalence of neonatal hypoglycemia and its risk factors were inconsistent in findings, and it is the first by its nature on prevalence of prolonged transitional hypoglycemia and associated factors in Ethiopia. The increased prevalence of prematurity and low birth weight make such a review vital for the formulation of prevention and management to neonates in specific and under-five children in general.

# Method and materials

The results of this review were reported based on the Preferred Reporting Items for Systematic Review and Meta-analysis (PRISMA) statement guidelines[17](**supplementary file 1)**. Data was extracted from 20-27 May 2023 for studies since 2020. The protocol is registered in the PROSPERO database (PROSPERO ID: CRD42023424953; https://www.crd.york.ac.uk/prospero/myprospero.

## Search strategy and information source

The adapted PICO format was used to explicitly review the literature and clarify the specifications of the inclusion and exclusion criteria. The adapted PICO comprises population (P), exposure (E), outcome (O), and context (setting as described below).

a. **Population**: all neonates irrespective of gestational age and birth weight
b. **Exposure**: Associated factors, predictors, risk factors
c. **Outcome**: prolonged transitional neonatal hypoglycemia
d. **Context (Setting):** Ethiopia.

Hence, by using this adapted PICO format, the following review questions were developed for the search for data.

1. What is the national incidence of prolonged transitional neonatal hypoglycemia in Ethiopia?
2. What are the factors associated with prolonged transitional neonatal hypoglycemia in Ethiopia?

Based on the aforementioned review PICO format and questions, primary studies were identified from MEDLINE, Scopus, Embase, Science Direct, Google Scholar, the African Journal Online, the Addis Ababa University repository, and the Haramay University repository.

The core search terms and phrases used were “prevalence”, “incidence”, “epidemiology”, “proportion”, “magnitude”, “burden”, “persistent”, “late newborn”, “recurrent”, “low blood glucose”, “predictors”, “risk factors”, “associated factors” and “Ethiopia”. The search strategies were developed using different Boolean operators. Notably, to fit the advanced MEDLINE database, the following search strategy was applied on April 8, 2023: [(prevalence[MeSH Terms]) OR incidence[MeSH Terms]) OR proportion [MeSH Terms]) OR epidemiology[MeSH Terms]) OR magnitude[MeSH Terms]) OR burden[MeSH Terms]) AND predictors [MeSH Terms]) OR risk factors [MeSH Terms]) OR associated factors [MeSH Terms]) OR neonatal hypoglycemia [MeSH Terms]) OR newborn hypoglycemia [MeSH Terms]) OR low blood glucose in newborns [MeSH Terms]) OR low random blood glucose level in neonates [MeSH Terms]) AND (Ethiopia)].

## Study selection

Primary studies downloaded and retrieved were exported to Mendeley Desktop 1.19.8 reference manager software to remove duplicate studies. The study selection process was held in two stages. First, the title and abstract were screened, and second, a full-text review was performed. Two independent reviewers (DKM and AK) screened the title and abstract. Disagreements were resolved based on established article selection criteria, and the third author (SDK) resolved disagreements to decide whether the study was eligible for inclusion if the quality appraisal score was 5 and more out of 9. Two independent authors subsequently reviewed the abstracts and full texts of the eligible articles.

# Eligibility criteria

**The inclusion criteria for patients were as follows**: articles that reported the prevalence of prolonged transitional neonatal hypoglycemia in general and/or at least one associated factor and published in English and studies conducted only in Ethiopia.

**The exclusion criteria for patients were as follows:** forms of hyperinsulinism or other diseases causing hypoglycemia such as inborn errors of metabolism were excluded. Moreover, articles without full-text information and qualitative studies were not reviewed.

# Critical appraisal and quality assessment

We used the quality appraisal criteria of the Joanna Briggs Institute (JBI)[18]. The tool consists of nine major items. The first item is appropriate for the sample frame. The second is the appropriate sampling technique. The third is the adequacy of the sample size. The fourth is a description of the study subjects and settings. The fifth is enough coverage of the data analysis. The sixth is the validity of the method for identifying the condition. The seventh item is a standard and reliable measurement for all participants. The eighth is the appropriateness of the statistical analysis. The last item is adequacy and management of the response rate. Studies were considered low risk when they fit 5 or more quality assessment checklists. Two independent authors (TMA and KA) appraised the quality of the studies. Disagreements were resolved by the interference of a third reviewer (SDK). The quality scores for the primary studies were reported (**supplementary file 2).**

# Data extraction

The data were determined to be extracted based on two criteria: 1. Clear and consistent operational definitions for the dependent variable (persistent neonatal hypoglycemia) and 2. These variables are statistically associated with the outcome variable reported by the AOR. Two authors extracted the data using the standardized format of the MS Excel spreadsheet (**Supplementary file 3**). The name of the first author and year, study region, study area, study design, random blood glucose (RBS) cutoff point, sample size, prevalence, and odds of associated factors were extracted from 20-27 May 2023. Whenever variations were observed, the phase was repeated. If discrepancies between the data were resolved, a third reviewer was consulted. After reporting the inconsistency of the data in the primary study, data transformation was conducted.

# Outcome measures

**Prolonged transitional neonatal hypoglycemia (PTNHG):** Neonates with a random blood sugar (RBS) concentration <47 mg/dL at 48-72 hours after birth at the bedside by a random blood glucose analysis from a capillary sample [3,4].

# Statistical analysis

The required data were collected using the Microsoft Excel 2013 workbook. Then, STATA version 17 statistical software was used for the meta-analysis. Publication bias was objectively checked using Egger's regression test analysis[19]. The heterogeneity of the studies was quantified using the I-squared statistic [20]. Both pooled prevalence analysis and pooled effect of associated factors were conducted using a weighted inverse variance random-effects model [21]. Subgroup analysis was performed by diagnostic method and sample size to overcome the inflation of the pooled effect from the inclusion of studies.

# Results

## Literature search

The search strategy retrieved 64 articles electronically and manually, of which 18 duplicate articles were identified and removed. Among the 46 screened articles, 46 were from PubMed (n=19), Google Scholar (n=23), and other databases of the Addis Ababa University repository (n=2), the Haramaya University database (n=1), and the manuscript (n=1). The full texts of nineteen articles were reviewed, and eight studies were included in the review and meta-analysis (**figure 1**).

## Characteristics of the included studies

Eight cross-sectional studies with a pooled sample size of 3,686 neonates were included in this meta-analysis. Two studies were found in each part of Ethiopia, namely, at Central, Eastern, Northwest and Northern [5,22–28]. Four studies used an RBS cutoff of <40 mg/dl for the diagnosis of prolonged transitional neonatal hypoglycemia [22,23,27,28]. The remaining four studies used an RBS cutoff point of less than 47 mg/dl for the diagnosis of PTNHGs [5,25,26,29]. The maximum sample size was 769, and the minimum sample size was 196 among the reviewed studies [27,29]. The studies included in this systematic review and meta-analysis had no considerable risk. Therefore, all the studies were considered. We assessed the studies with a JBI quality appraisal checklist for systematic review [30]. According to this appraisal checklist, none of the included studies were of poor quality and had a quality score ≥6 (**Table 1).**

**Table 1.** Characteristics and quality status of the studies included, 2023.

| Author and year | Parts of Ethiopia | Capillary blood RBS cut point | Sample size | Incidence (%) of PNHG | JBI quality status |
|---|---|---|---|---|---|
| Bogale et al., 2021 | Northern (UOG) | <40 mg/dl | 399 | 13.53 | Low risk |
| Demis et al., 2022 | North-central (DTCSH) | <47 mg/dl | 400 | 23.50 | Low risk |
| Fantahun & | Central St. | <47 mg/dl | 196 | 25.00 | Low risk |

| | | | | | |
|---|---|---|---|---|---|
| Nurussen, 2021 | (PHMMC) | | | | |
| Kasaye et al., 2021 | Central (Addis Ababa) | <40 mg/dl | 317 | 20.82 | Low risk |
| Sertsu et al., 2022 | Eastern (HFSTH) | <40 mg/dl | 698 | 21.20 | Low risk |
| Yohanneset al., 2021 | Eastern (Harar and Dire Dawa) | <47 mg/dl | 640 | 14.68 | Low risk |
| Chanie et al., 2023 | North-central (DTCSH) | <47 mg/dl | 267 | 23.60 | Low risk |
| Demisse et al., 2017 | Northern (UOG) | <40 mg/dl | 769 | 18.46 | Low risk |

**UOG=University of Gondar, St. PHMMC=Saint Paul's Millennium Medical College, DTCSH=Debre Tabor Comprehensive Specialized Hospital, Data extracted date was on 20-27/05/2023, Data extractors were SDK, TMA, DK and KA**

# Meta-analysis

The pooled prevalence of prolonged transitional neonatal hypoglycemia was reviewed, and the absence of publication bias was assessed with Egger's regression test analysis (p = 0.068), which showed no publication bias (**Table 2**).

**Table 2** Egger's test indicating no publication bias.

| **Std.-Eff.** | **Coef.** | **Std. Err** | **t** | **p>t** | **95% confidence interval** | |
|---|---|---|---|---|---|---|
| **Slope** | 5.21962 | 2.177999 | 2.40 | 0.054 | -.1097508 | 10.54899 |
| **Bias** | 9.567008 | 4.312786 | 2.22 | **0.068** | -.9859986 | 20.12001 |

The pooled prevalence of PTNHG from eight studies [22–29] was 19.71 (95% CI: 16.85-22.56; $I^2$=79.20, P<0.001)(**Figure 2**).

## Subgroup analysis

Subgroup analysis was performed by using the sample size and the RBS cutoff point to diagnose prolonged transitional neonatal hypoglycemia (PTNHG). However, there was no effect on reducing the heterogeneity of the heterogeneity ($I^2$). According to the three studies of pooled effects, the prevalence with sample sizes >400 was 18% (15-22), and the prevalence with sample sizes ≤ 400 was 21% (17-26). The pooled effect of the RBS cutoff point for the diagnosis of PTNHG by (<40 mg/dl) were 18% (15-22), and by (<47 mg/dl) was 21% (16-27) (**Table 3)**.

**Table 3**. Subgroup analysis by sample size category and random blood sugar cutoff point for PTNHG and associated factors in Ethiopia, 2023.

| **Subgrouping by variables** | **Authors** | **Pooled prevalence at (95% CI)** | **$I^2$(P value)** |
|---|---|---|---|
| Sample size >400 | [23,24,27] | 18(15-22) | 78%(0.01) |
| Sample size ≤400 | [22,25,26,28,29] | 21(17-26) | 79.67%(0.00) |
| RBS<40 mg/dL | [22,23,27,28] | 18(15-22) | 73.83%(0.01) |
| RBS<47 mg/dL | [24–26,29] | 21(16-27) | 85.34%(0.00) |

**Data extracted date was on 20-27/05/2023, Data extractors were SDK, TMA, DK and KA**

## Factors associated with prolonged transitional neonatal hypoglycemia

Based on this review, the factors associated with persistent neonatal hypoglycemia identified by pooling two or more studies were preterm birth, neonatal hypothermia, neonatal birth from a chronic diabetic mellitus mother, breastfeeding initiation after an hour, and pathological jaundice.

According to previous studies [23,26], neonates who were delivered prematurely were three more likely to develop prolonged transitional neonatal hypoglycemia than neonates who were born at term in Ethiopia (AOR=3.31; 95% CI=2.57-4.04)**.** A pooled analysis of four studies

[23,25,26,29] revealed that neonates with hypothermia were 3.41 times more likely (AOR=3.41; 95% CI=2.19-4.62) to develop PTNHG than their normotensive counterparts were**.** There were nearly five times more neonates whose mothers had known chronic DM (AOR=4.71; 95% CI=2.15-7.26) than did those whose mothers did not have known chronic DM [22,23,25].

According to previous studies [22,23], neonates who had been breastfeeding after an hour were three times more likely to be hypoglycemic than neonates who had been breastfeeding within an hour (AOR=3.26; 95% CI=2.03-4.49)**.** By pooling of two studies[24,26] revealed that the incidence of pathological jaundice in neonates was almost twofold-fold greater (AOR=1.70; 95% CI=1.21-2.99) than that in their counterparts without pathological jaundice**.** One study [23] revealed that the odds of neonatal sepsis were nearly three times greater than that of neonates with no sepsis, and the odds of neonates with very low birth weight (VLBW) were four times greater than those of neonates with normal birth weight(NBW) (AOR=2.61; 95% CI=1.68-3.54) and (AOR=4.01; 95% CI=1.85-9.87), respectively (**Table 4**).

**Table 4**: The pooled effect of factors associated with PNHG in Ethiopia, 2023.

| **Factors** | **Author and year** | **Pooled OR (95% CI)** | **$I^2$(P value)** |
|---|---|---|---|
| Preterm birth | (Sertsu et al., 2022) | 3.31(2.57-4.04) | 0.01%(0.001) |
| | (Demis et al., 2022) | | |
| Neonatal Hypothermia | (Sertsu et al., 2022) | 3.41(2.19-4.62) | 97.96%(0.001) |
| | (Fantahun and Nurussen, 2021) | | |
| | (Chanie et al., 2023) | | |
| | (Demis et al., 2022) | | |
| Infants of diabetic mother (IDM) | (Sertsu et al., 2022) | 4.71(2.15-7.26) | 94%(0.001) |
| | (Chanie et al., 2023) | | |
| | (Kasaye et al., 2021) | | |
| Breastfeeding after one hour | (Sertsu et al., 2022) | 3.26(2.03-4.49) | 52.80%(0.001) |
| | (Kasaye et al., 2021) | | |
| Pathological jaundice | (Demis et al., 2022) | 2.37(1.91-2.84) | 0.01%(0.001) |
| | (Yohannes et al., 2021) | | |

**Data extracted date was on 20-27/05/2023, Data extractors were SDK, TMA, DK and KA**

According to a previous study [25], for neonates with one or more episodes of seizure and asphyxia stage II or III HIE, PTNHG had odds of nearly five times and five times greater than

their counterparts (AOR=4.70; 95% CI=3.78-5.62) and (AOR=5.10; 95% CI=4.18-6.02), respectively. Other single studies (Kasaye, 2021) showed that neonates whose mothers had GDM or preeclampsia during their latest pregnancy were approximately eight or two times more likely to have GDM or preeclampsia, respectively (AOR=7.79; 95% CI=5.87-9.71) and (AOR=2.44; 95% CI=1.65-3.23), than their counterparts were. In contrast, a study by [26] revealed that neonates delivered via SVD were 28% less likely to develop PTNHG than were those delivered via CS (AOR=0.72; 0.35-1.79).

# Discussion

This systematic review and meta-analysis aimed to assess the pooled incidence of PTNHGs and associated factors in Ethiopia. The pooled prevalence was 19.71 (95% CI: 16.85-22.56), which was comparable to that reported in studies in Iran Tehran [31], China Shanghai [32] and the USA [33], which had a prevalence of 15.50% and 16.90%, respectively, and a range of 8-30%, respectively.

On the other hand, studies from Pakistan used RBS cutoff values <50 mg/dl [34], and on the basis of RBS cutoff values <40 mg/dl and 47 mg/dl [35], it was found that the incidence of neonatal hypoglycemia was much lower than that in this review (0.4% and 3.4-12.10%, respectively). This might be because this review utilized the operational definition of persistent neonatal hypoglycemia, and the aforementioned literature included all types of neonatal hypoglycemia. Moreover, the variation in sample size and socio-demographic variables might contribute to this difference.

According to our subgroup analysis based on the RBS cutoff for diagnosing PTNHG, the estimated pooled prevalence obtained by pooling the studies with an RBS cutoff<47 mg/dl was 21% greater (95% CI: 16-27) than that obtained by pooling the studies with an RBS cutoff <40 mg/dl 18% (95% CI: 15-22). A possible explanation might be the difference between the RBS cutoff points; therefore, <47 mg/dl is associated with a greater probability that more neonates would have RBS measurements within that range. This result is supported by studies in Israel in which the incidence of neonatal hypoglycemia substantially differed between the RBS cutoff <40 mg/dl and <47 mg/dl at 3.4% and 12.10%, respectively [35]. Similarly, there was a difference in the pooled estimate of PTNHG between the primary studies with a sample size >400 at 18%

(95% CI: 15-22) and those with ≤400 at 21% (95% CI: 17-26). This difference can be explained by the fact that studies with sample size variation affected the outcome of the data analysis. Hence, a sample size >400 had more strength in determining the true value statistically in the regression analysis than a sample size ≤400.

This meta-analysis revealed that preterm infants were 3 times more likely to develop PTNHG than term neonates were. This could be because preterm neonates are uniquely predisposed to hypoglycemia of any type and its related complications due to their limited glycogen and fat stores, through which body thermoregulation can be maintained. Premature neonates' inability to generate new glucose via gluconeogenesis pathways affects their glucose utilization in metabolic processes. This, in turn, requires greater metabolic demands due to having a relatively larger brain than term neonates because they are unable to respond to a counter regulatory mechanism to hypoglycemia compared to term neonates [23,36,37].

Similarly, another factor associated with PTNHGs was neonatal hypothermia, which increased the odds of PTNHG development by 3-fold compared with that of their counterparts with normothermic neonates. The scientific evidence was congruent with these findings in that neonatal hypothermia predisposes to neonatal hypoglycemia. Neonates exhibit a metabolic response to cooling that involves chemical but no shivering thermogenesis via the sympathetic nerve discharge of norepinephrine in brown fat. This reaction increases the metabolic rate and oxygen consumption 2-3-fold by activating glycogen stores, thereby causing transient hypoglycemia as a compensatory mechanism and persistent hypoglycemia as a disorder, as impaired body glucose hemostasis results in metabolic acidosis and increased risk of death [38–42].

Neonates who gave birth from mothers with chronic diabetes mellitus were nearly five times more likely to develop PTNHG than were those from mothers without chronic diabetes mellitus. The justification for this finding is that an infant of a diabetic mother (IDM) is more likely to have a period of low blood sugar shortly after birth and during the first few days of life.

This might be because neonates obtain more sugar than needed from the mother in the uterus before delivery. As such, neonates have higher insulin levels than needed after birth, and insulin then lowers their blood sugar level, allowing them to adjust to a normal RBS after birth for several days [43–45].

Regarding breastfeeding initiation time, the odds that neonates who initiated breastfeeding after an hour were approximately three times greater (AOR=3.26; 95% CI=2.03-4.49) of developing PTNHG than were those who breastfed within an hour after delivery [22,23]. This evidence, combined with the pooled ORs estimated, was strongly reflected in the results of a single study indicating that neonates who delivered via spontaneous vaginal delivery(SVD) were 72% less likely (AOR=0.72; -0.35-1.79) to develop PTNHG than were those who delivered via cesarean section delivery (CS) [26]. This is justified by the fact that breast milk is the ideal food for infants that protects against neonatal hypoglycemia. The initiation of breastfeeding within an hour is the mainstay for preventing persistent neonatal hypoglycemia, as per WHO recommendations. However, neonates who delivered via the CS were 2.01 times more likely to develop neonatal hypoglycemia. Hence, any cause that delays breastfeeding within one hour, including CS delivery, should be considered. A delay in breastfeeding results in neonatal hypoglycemia because breast milk provides all the energy and nutrients that the infant needs for the first months of life and continues to provide up to half or more of a child's nutritional needs [1,46–48].

On the other hand, neonates with pathological jaundice had approximately 2.37 greater odds (AOR=2.37; 95% CI=1.91-2.84) than did those without pathological jaundice [24,26]. These findings are comparable to those of other studies in that neonates with hyper-bilirubinemia were 1.81 and 1.93 times more likely to develop neonatal hypoglycemia than were those with normal bilirubin test values. In another study, 8.80% of neonates treated with phototherapy developed neonatal hypoglycemia [48–50].

This might be because the relationship between neonatal hypoglycemia and neonatal hyper-bilirubinemia in pathological jaundice might be mutually influenced by the fact that OGTT 2-h glucose levels affect neonatal hypoglycemia. Abnormal postprandial glucose levels suggested impaired glucose tolerance and β-cell dysfunction on a physiological basis due to jaundice. As a result, neonates with jaundice are affected by the physiological process of gluconeogenesis to maintain a norm-glycemic state.

According to the findings from a single primary study, patients diagnosed with early neonatal sepsis had greater odds of developing neonatal hypoglycemia (AOR=2.61; 95% CI=1.68-3.54). These findings were supported by the findings of the literature that early onset of neonatal sepsis

(EONS) with neonatal hypoglycemia was approximately 8.7% and 9.6%, respectively, among neonates admitted for sepsis.

This is because neonates with neonatal sepsis tend to refuse to breastfeed, resulting in low blood sugar. Similarly, increased metabolic demand is caused by low glucose levels in sepsis and hypothermia [51,52]. According to a previous study [25], for neonates with one or more episodes of seizure and asphyxia stage II or III HIE, PTNHG had odds of nearly five times and five times greater than their counterparts (AOR=4.70; 95% CI=3.78-5.62) and (AOR=5.10; 95% CI=4.18-6.02), respectively. This increased risk for hypoglycemia might be due to a lack of storage in combination with high insulin levels, which predisposes patients to hypoglycemia. Perinatal asphyxia (PNA) and perinatal seizures secondary to PNA increase the risk of hyper-insulinism in the neonatal period because of the use of anaerobic metabolism to maintain blood glucose concentrations. Anaerobic metabolism is used to maintain blood glucose concentrations [53]. Finally, a study [22] showed that neonates with gestational diabetes mellitus (GDM) or preeclampsia during their latest pregnancy were approximately eight times and two times more likely, respectively (AOR=7.79; 95% CI=5.87-9.71) and (AOR=2.44; 95% CI=1.65-3.23), than their counterparts were. This can be explained by the fact that the most common complication in infants born to GDM mothers is hypoglycemia. This is due to glucose freely passing through the placenta; therefore, maternal hyperglycemia associated with GDM results in elevated glucose levels in the fetus, causing excess fetal insulin production, termed hyper-insulinism[54].

# Conclusion

Persistent neonatal hypoglycemia is quite common in Ethiopia. The pooled prevalence estimate of PTNHG from eight studies was nearly ten percent. Neonates who delivered prematurely, neonates with hypothermia, neonates of diabetic mothers (IDM), neonates who had not been breastfeeding after an hour and neonates with pathological jaundice had higher odds of developing PNHG than did their counterparts. According to single primary studies, neonatal sepsis, very low birth weight(VLBW), neonates with one or more episodes of seizure, and asphyxia stage II or III HIE, neonates of GDM mothers had higher odds of developing neonatal hypoglycemia. In contrast, neonates delivered by SVD were protected against neonatal hypoglycemia. As per this review, the pooled prevalence of PNHGs was significant. Several associated factors were pooled so that intervention strategies would be needed to reduce and

prevent their occurrence while treating neonates in neonatal intensive care units in Ethiopia. Thus, the team of this review strongly advised health care professionals, including nurses, doctors, and others working in the NICU, to monitor and regulate these and other determining variables that predispose patients to neonatal hypoglycemia, particularly the persistent type.

### Contribution of the Review

Given the national prevalence and associated factors of persistent neonatal hypoglycemia (PNHG), evidence-based decisions will be used in the prevention and treatment of persistent neonatal hypoglycemia in health care settings. Hence, healthcare professionals, others who work in the neonatal intensive care unit (NICU) and the directors of those services can adopt this finding by incorporating it as the priority concern in neonatal care standards in Ethiopia.

## Availability of data

The data was uploaded as supplementary information.

## Ethics approval

Not applicable.

## Conflicts of interest

All authors declared no conflicts of interests

## Author contributions

**Conceptualization:** Solomon D. Kebede

**Data curation:** Kindu Agmas

**Formal analysis and methodology:** Solomon D. Kebede

**Supervision and commenting:** Demewoz Kefale and Amare Kassaw

**Writing original draft:** Solomon D. Kebede and Tigabu M. Aytenew

**Writing, review and editing:** Solomon D. Kebede

## Funding

The authors received no specific funding for this work.

## Patient and public involvement

Patient and/or the public were not involved in the design, or conduct, or reporting, or dissemination plans of this research.

## Abbreviations

**CS**: Cesarean Section, **GDM**: Gestational Debates Mellitus, **HIE**: Hypoxic Ischemic Encephalopathy, **IDM**: Infant of Diabetics' Mother, **PTNHG:** Prolonged Transitional Neonatal Hypoglycemia, **SGA**: Small for Gestational Age, **SVD**: Spontaneous Vaginal Delivery, **VLBW**: Very Low Birth Weight

## References

1. WHO. Guideline:Protecting, promoting and supporting breastfeeding in facilities providing maternity and newborn services. World Health OrganizationWHO. 2017. Available: https://apps.who.int/iris/bitstream/handle/10665/259386/9789241550086-eng.pdf
2. Cornblath M, Hawdon JM, Williams AF, Aynsley-Green A, Ward-Platt MP, Schwartz R, et al. Controversies regarding definition of neonatal hypoglycemia: Suggested operational thresholds. Pediatrics. 2000;105: 1141–1145. doi:10.1542/peds.105.5.1141
3. Abramowski A, Ward R, Hamdan AH. Neonatal hypoglycemia. StatPearls [Internet]. StatPearls Publishing; 2021.
4. Thompson-Branch A, Havranek T. Neonatal hypoglycemia. Pediatr Rev. 2017;38: 147–157.
5. Paul S, Yismaw Fantahun B. Prevalence and Risk factors of Hypoglycaemia in Neonates at: A Cross Sectional Study. 2020; 1–15.
6. Frank-Briggs AI, Ojule AC, Nkanginieme KE. Neonatal hypoglycaemia: prevalence and clinical manifestations in Port Harcourt, Nigeria. Port Harcourt Med J. 2008;2: 166–170.
7. Shah R, Harding J, Brown J, McKinlay C. Neonatal glycaemia and neurodevelopmental

outcomes: a systematic review and meta-analysis. Neonatology. 2019;115: 116–126.

8. Mahajan G, Mukhopadhyay K, Attri S, Kumar P. Neurodevelopmental Outcome of Asymptomatic Hypoglycemia Compared With Symptomatic Hypoglycemia and Euglycemia in High-Risk Neonates. Pediatr Neurol. 2017;74: 74–79. doi:https://doi.org/10.1016/j.pediatrneurol.2017.05.028

9. Sultan OM. The Hidden Burden of Hypoglycemia in Neonatal Mortality in Tanzania-The Case of Saint Francis Hospital, Ifakara. IMTU Med J. 2012;3: 34–39.

10. Kush ML, Baschat AA. Fetal responses to placental insufficiency. Textbook of Perinatal Medicine. CRC Press; 2006. pp. 1021–1038.

11. Menni F, De Lonlay P, Sevin C, Touati G, Peigné C, Barbier V, et al. Neurologic outcomes of 90 neonates and infants with persistent hyperinsulinemic hypoglycemia. Pediatrics. 2001;107: 476–479.

12. Tam EWY, Widjaja E, Blaser SI, MacGregor DL, Satodia P, Moore AM. Occipital lobe injury and cortical visual outcomes after neonatal hypoglycemia. Pediatrics. 2008;122: 507–512.

13. McKinlay CJD, Alsweiler JM, Anstice NS, Burakevych N, Chakraborty A, Chase JG, et al. Association of neonatal glycemia with neurodevelopmental outcomes at 4.5 years. JAMA Pediatr. 2017;171: 972–983.

14. Lucas A, Morley R, Cole TJ. Adverse neurodevelopmental outcome of moderate neonatal hypoglycaemia. BMJ. 1988;297: 1304–1308. doi:10.1136/bmj.297.6659.1304

15. You D, Hug L, Ejdemyr S, Idele P, Hogan D, Mathers C, et al. Global, regional, and national levels and trends in under-5 mortality between 1990 and 2015, with scenario-based projections to 2030: a systematic analysis by the UN Inter-agency Group for Child Mortality Estimation. Lancet. 2015;386: 2275–2286.

16. Gebremichael SG, Fenta SM. Under-Five Mortality and Associated Risk Factors in Rural Settings of Ethiopia: Evidences from 2016 Ethiopian Demographic and Health Survey. Adv Public Heal. 2020;2020. doi:10.1155/2020/8430246

17. Moher D, Shamseer L, Clarke M, Ghersi D, Liberati A, Petticrew M, et al. Preferred reporting items for systematic review and meta-analysis protocols (PRISMA-P) 2015 statement. Syst Rev. 2015;4: 1–9.

18. Peters MDJ, Godfrey CM, McInerney P, Soares CB, Khalil H, Parker D. The Joanna Briggs Institute reviewers' manual 2015: methodology for JBI scoping reviews. 2015.

19. Rothstein HR, Sutton AJ, Borenstein M. Publication bias in meta-analysis. Publ bias meta-analysis Prev Assess Adjust. 2005; 1–7.

20. Ioannidis JPA. Interpretation of tests of heterogeneity and bias in meta-analysis. J Eval Clin Pract. 2008;14: 951–957.

21. Borenstein M, Hedges L V, Higgins JPT, Rothstein HR. A basic introduction to fixed-effect and random-effects models for meta-analysis. Res Synth Methods. 2010;1: 97–111.

22. KASAYE F. COLLEGE OF HEALTH SCIENCE SCHOOL OF NURSING AND MIDWIFERY DEPARTMENT OF NURSING PREVALENCE AND PREDICTORS OF HYPOGLYCEMIA AMONG NEONATES ON ADMISSION TO NEONATAL INTENSIVE CARE UNIT IN SELECTED PUBLIC HOSPITALS , PRINCIPAL INVESTIGATOR : FIKIRTE KASAYE (. 2021.

23. Sertsu A, Nigussie K, Eyeberu A, Tibebu A, Negash A, Getachew T, et al. Determinants of neonatal hypoglycemia among neonates admitted at Hiwot Fana Comprehensive Specialized University Hospital, Eastern Ethiopia: A retrospective cross-sectional study. SAGE Open Med. 2022;10. doi:10.1177/20503121221141801

24. YOHANNES M. Magnitude of Jaundice and Associated Factors among Neonates Admitted to Neonatal In_tensive Care Unit in Eastern Ethiopia. Front Neurosci. 2021;14: 1–13.

25. Chanie ES, Shiferaw SM, Feleke DG, Bantie B, Moges N, Tasew SF, et al. Maximizing the detection rate of hypoglycemia among preterm neonates admitted in Neonatal intensive care unit in Ethiopia, 2021. Sci Rep. 2023;13: 1–7. doi:10.1038/s41598-023-29112-y

26. Demis S, Munye T, Agmas K, Necho W, Moges N. Incidence of Persistent Neonatal Hypoglycemia and Associated Factors among Neonatal Intensive Care Unit Admissions in South Gondar Public Hospitals , Northcentral Ethiopia : a prospective cross sectional study. unpublished. 2022; 1–24.

27. Demisse AG, Alemu F, Gizaw MA, Tigabu Z. Patterns of admission and factors associated with neonatal mortality among neonates admitted to the neonatal intensive care unit of University of Gondar Hospital, Northwest Ethiopia. Pediatr Heal Med Ther. 2017;Volume 8: 57–64. doi:10.2147/phmt.s130309

28. Bogale MT, Bogale WA, Kassie DG, Woldesellassie A, Tamiru AT. Factors associated with neonatal hyperbilirubinemia in case files of all admitted inborn and outborn neonates in Northwest Ethiopia in 2019. Iran J Neonatol. 2021;12: 46–53. doi:10.22038/ijn.2020.49279.1859

29. Fantahun B, Nurussen I. Prevalence and risk factors of neonatal hypoglycaemia at st. Paul's Hospital Millennium Medical College, Ethiopia. Pediatr Child Heal. 2021;26: 13. Available: https://www.researchgate.net/profile/Bereket-Fantahun-2/publication/358834078_ORIGINAL_ARTICLE_PREVALENCE_AND_RISK_FACTORS_OF_NEONATAL_HYPOGLYCAEMIA_AT_ST_PAUL'S_HOSPITAL_MILLENNIUM_MEDICAL_COLLEGE_ETHIOPIA/links/6217e57e6738db292ac385ac/ORIGINAL-ARTICLE-

30. Briggs J. Checklist for Systematic Reviews and Research Syntheses. The Joanna Briggs Institute. 2017. p. 7. Available: http://joannabriggs.org/research/critical-appraisal-tools.htmlwww.joannabriggs.org%0Awww.joannabriggs.org

31. Dashti N, Einollahi N, Abbasi S. NEONATAL HYPOGLYCEMIA : PREVALENCE AND CLINICAL MANIFESTATIONS IN TEHRAN CHILDREN ' S HOSPITAL. 2007;23.

32. Zhou W, Yu J, Wu Y, Zhang H. Hypoglycemia incidence and risk factors assessment in hospitalized neonates neonates. 2015;7058: 6–10. doi:10.3109/14767058.2014.918599

33. Alemu BT, Olayinka O, Baydoun HA, Hoch M, Elci MA. Neonatal Hypoglycemia in Diabetic Mothers : a Systematic Review. 2017;21: 42–53.

34. Najati N, Saboktakin L. Prevalence and underlying etiologies of neonatal hypoglycemia. Pakistan Journal of Biological Sciences. 2010. pp. 753–756. doi:10.3923/pjbs.2010.753.756

35. Bromiker R, Perry A, Kasirer Y, Einav S, Klinger G. Early neonatal hypoglycemia : incidence of and risk factors . A cohort study using universal point of care screening. J Matern Neonatal Med. 2019;32: 786–792. doi:10.1080/14767058.2017.1391781

36. Sharma A, Davis A, Shekhawat PS. Hypoglycemia in the preterm neonate: etiopathogenesis, diagnosis, management and long-term outcomes. Transl Pediatr. 2017;6: 335–348. doi:10.21037/tp.2017.10.06

37. Goode RH, Rettiganti M, Li J, Lyle RE, Whiteside-Mansell L, Barrett KW, et al. Developmental outcomes of preterm infants with neonatal hypoglycemia. Pediatrics. 2016;138. doi:10.1542/peds.2016-1424

38. Yitayew YA, Aitaye EB, Lechissa HW, Gebeyehu LO. Neonatal Hypothermia and Associated Factors among Newborns Admitted in the Neonatal Intensive Care Unit of Dessie Referral Hospital, Amhara Region, Northeast Ethiopia. Int J Pediatr. 2020;2020: 3013427. doi:10.1155/2020/3013427

39. Strauch, Barry S.Felig, PhilipBaxter JD. 2023. 2023.

40. Agardh CD, Smith ML, Siesjö BK. The influence of hypothermia on hypoglycemia-induced brain damage in the rat. Acta Neuropathol. 1992;83: 379–385. doi:10.1007/BF00713529

41. Kedes LH, Field JB. Hypothermia. N Engl J Med. 1964;271: 785–787. doi:10.1056/NEJM196410082711509

42. Tran C, Gariani K, Herrmann FR, Juan L, Philippe J, Rutschmann OT, et al. Hypothermia is a frequent sign of severe hypoglycaemia in patients with diabetes. Diabetes Metab. 2012;38: 370–372. doi:10.1016/j.diabet.2012.03.005

43. Kallem VR, Pandita A, Pillai A. Infant of diabetic mother: what one needs to know? J Matern Neonatal Med. 2020;33: 482–492. doi:10.1080/14767058.2018.1494710

44. Mormile R. Neonates of diabetic mothers: The starting point for developing novel therapeutic approaches to ischemic heart and brain? Med Hypotheses. 2016;96: 75–77. doi:10.1016/j.mehy.2016.09.009

45. Opara PI, Jaja T, Onubogu UC. Morbidity and mortality amongst infants of diabetic mothers admitted into a special care baby unit in Port Harcourt, Nigeria. Ital J Pediatr. 2010;36: 2–7. doi:10.1186/1824-7288-36-77

46. Ekubay M, Berhe A, Yisma E. Initiation of breastfeeding within one hour of birth among mothers with infants younger than or equal to 6 months of age attending public health institutions in Addis Ababa, Ethiopia. Int Breastfeed J. 2018;13: 1–7. doi:10.1186/s13006-018-0146-0

47. Karim F, Masum Billah SK, Chowdhury MAK, Zaka N, Manu A, Arifeen S El, et al. Initiation of breastfeeding within one hour of birth and its determinants among normal vaginal deliveries at primary and secondary health facilities in Bangladesh: A case-observation study. PLoS One. 2018;13: 1–15. doi:10.1371/journal.pone.0202508

48. He J, Song J, Zou Z, Fan X, Tian R, Xu J, et al. Association between neonatal hyperbilirubinemia and hypoglycemia in Chinese women with diabetes in pregnancy and influence factors. Sci Rep. 2022;12: 16975. doi:10.1038/s41598-022-21114-6

49. Kallem VR, Pandita A, Pillai A. Infant of diabetic mother: what one needs to know? J Matern neonatal Med Off J Eur Assoc Perinat Med Fed Asia Ocean Perinat Soc Int Soc Perinat Obstet. 2020;33: 482–492. doi:10.1080/14767058.2018.1494710

50. Salman M, Rathore H, Arif S, Ali R, Khan AA, Nasir M. Frequency of Immediate Neonatal Complications (Hypoglycemia and Neonatal Jaundice) in Late Preterm and Term Neonates. Cureus. 2021;13: e12512. doi:10.7759/cureus.12512

51. Islam MS, Mia MAH, Akhter KR, Haque M, Malik M. Glycemic Status and its Effect in Neonatal Sepsis in a Tertiary Care Hospital. Bangladesh J Child Heal. 2017;40: 21–25. doi:10.3329/bjch.v40i1.31551

52. Ellahony D, El-Mekkawy M, A. Issa AHR. Prevalence of glucose level abnormalities in neonatal sepsis and its association with mortality. Menoufia Med J. 2020;33: 127. doi:10.4103/mmj.mmj_216_18

53. Sweet CB, Grayson S, Polak M. Management strategies for neonatal hypoglycemia. J Pediatr Pharmacol Ther JPPT Off J PPAG. 2013;18: 199–208. doi:10.5863/1551-6776-18.3.199

54. Voormolen DN, de Wit L, van Rijn BB, DeVries JH, Heringa MP, Franx A, et al. Neonatal Hypoglycemia Following Diet-Controlled and Insulin-Treated Gestational Diabetes Mellitus. Diabetes Care. 2018;41: 1385–1390. doi:10.2337/dc18-0048

# Supporting information

**S1 Supplementary file 1**. PRISMA checklist

**S2 Supplementary file 2**. JBI quality appraisal criteria

**S3 Supplementary file 3.** Data of the review and meta-analysis

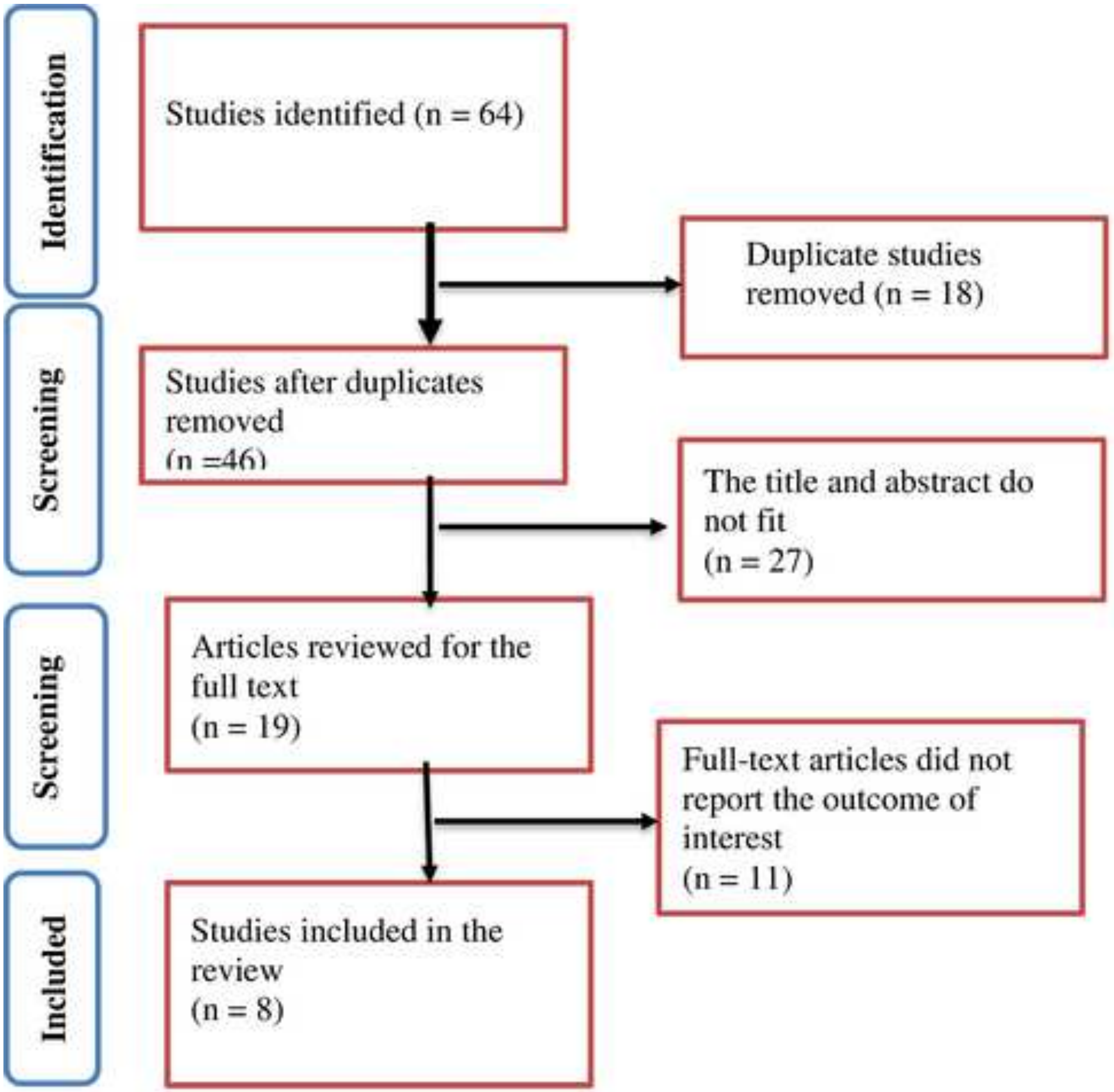


**Fig 1.** An adapted PRISMA 2020 flow diagram for new systematic reviews that include searches of databases, registers, and other sources in Ethiopia

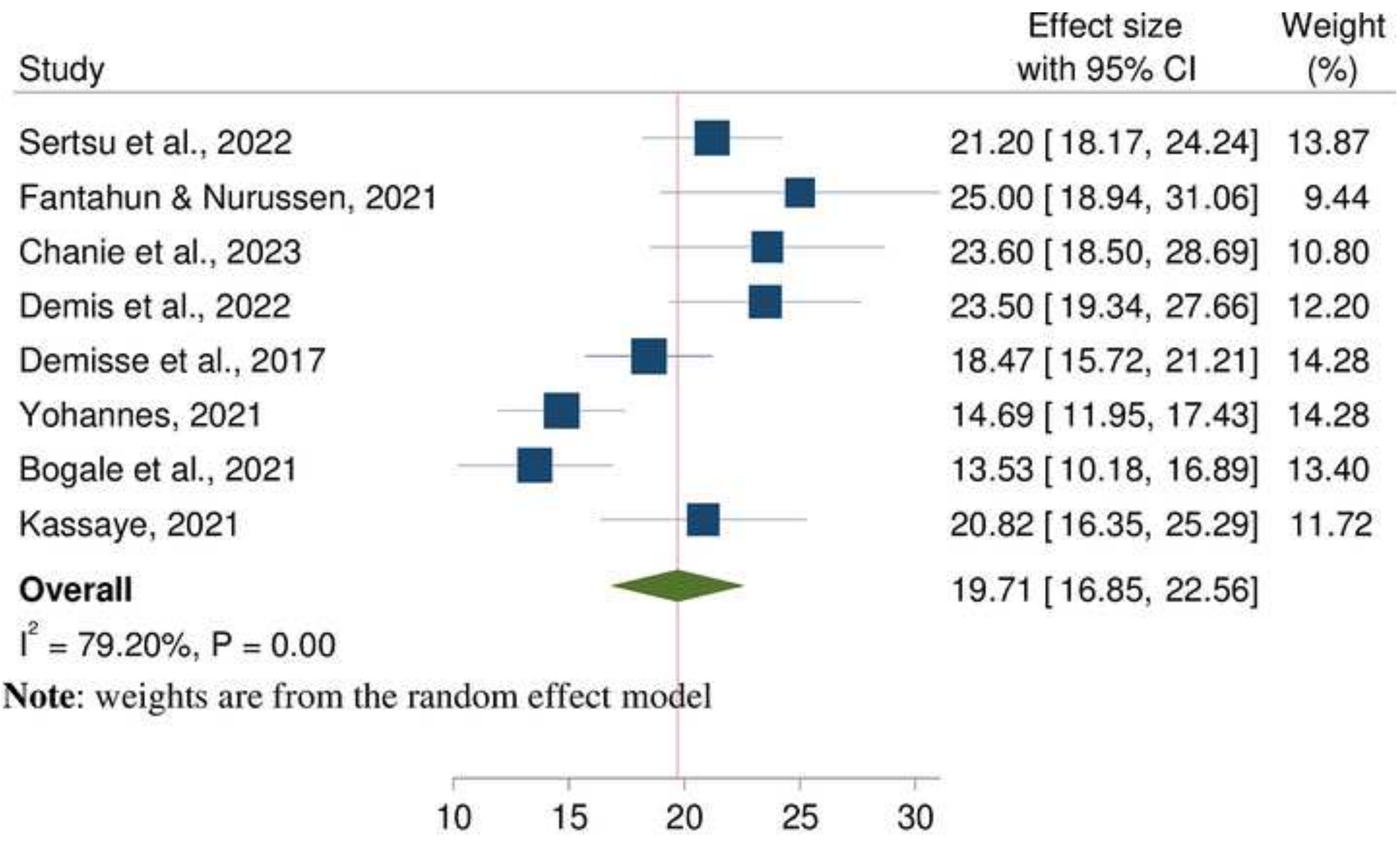


**Figure 2**. Forest plot for the pooled prevalence of PNHG in Ethiopia, 2023.

PRISMA checklist

Click here to access/download
**Supporting Information**
S 1.DOCX

JBI quality checklist

Click here to access/download
**Supporting Information**
S 2.DOCX

Click here to access/download
**Supporting Information**
Raw dat for PTNHG in Ethiopia.xlsx

# Prevalence of prolonged transitional neonatal hypoglycemia and associated factors in Ethiopia: A systematic review and meta-analysis

Formatted: Font: 18 pt, Font color: Red

Formatted: Font: 18 pt

**Solomon Demis Kebede[1], Amare Kassaw[1], Tigabu Munye Aytenew[2], Kindu Agmas[3], Demewoz Kefale[1]**

[1] Department of Pediatrics and Child Health Nursing, College of Health Sciences, Debre Tabor University, Debre Tabor, Ethiopia

[2] Department of Nursing, College of Health Sciences, Debre Tabor University, Debre Tabor, Ethiopia

[3] Departments of Pediatrics and Child Health, Debre Tabor Comprehensive Specialized Hospital, Debre Tabor, Ethiopia

***Correspondence**: solomondemis@gmail.com, mob. +251930713008

# Abstract

Formatted: Font: 16 pt

**Introduction:** Most neonates have transient hypoglycemia, which responds to treatment and is associated with a good prognosis compared with prolonged hypoglycemia. Hypoglycemia occurring after 48 hours of age is termed prolonged transitional hypoglycemia and results in abrupt injury and neurodevelopmental defects. **Method:** A weighted inverse-variance random effects model was used. Variations across the studies were checked using a forest plot, $I^2$ statistics, and Egger's test. A random blood sugar (RBS) concentration <47 mg/dL at 48-72 hours after birth was used to indicate prolonged transitional neonatal hypoglycemia (PTNHG). **Results**: Eight studies with a sample of 3686 neonates were included. The pooled prevalence of prolonged PTNHG was 19.71% (95% CI: 16.85-22.56); I2=79.20, P<0.001). By subgroup analysis from sample sizes > 400 and ≤ 400, PTNHG was found to be comparable at 18% (15-22) and 21% (17-26), respectively. On the other hand, when using RBS cutoffs <47 mg/dL and <40 mg/dL, the PTNHGs were relatively similar at 21% (16-27) and 18% (15-22), respectively. Preterm birth (AOR=3.31; 95% CI=2.57-4.04), hypothermia (AOR=3.41; 95% CI=2.19-4.62), infant of diabetes mother(IDM) (AOR=4.71; 95% CI=2.15-7.26), breastfeeding initiation after an hour (AOR=3.26; 95% CI=2.03-4.49), and pathological jaundice (AOR=2.37; 95% CI=1.91-2.84) were significantly associated with PTNHG. **Conclusion:** Nearly one-fifth of the neonates were in a state of hypoglycemia during their hospitalization. Fortunately, the factors were easily modifiable. Prioritization of the problem in current national NICU hospital practice guidelines, initiation of early breastfeeding, particularly for cesarean section (CS) delivery and ~~the infant of diabetes mother (~~IDM~~)~~ would reduce the burden.

**Prospero ID**: CRD42023424953.

**Keywords**: prevalence of neonatal hypoglycemia, prolonged transitional neonatal hypoglycemia, persistent neonatal hypoglycemia, incidence of hypoglycemia, newborn hypoglycemia

# Introduction

Neonatal hypoglycemia is defined as low blood glucose concentration below 40-50 mg/dL as per World Health Organization (WHO) and American Academy of Pediatrics (AAP)(1,2). There are two types of neonatal hypoglycemia: transient and prolonged. Most neonates have transient hypoglycemia, which responds to treatment and is associated with a good prognosis compared with persistent hypoglycemia. Hypoglycemia that occurs after 48 hours of age is termed prolonged transitional hypoglycemia, the clinical manifestations of which are generated in the short term, with abrupt injury and neurodevelopmental sequelae occurring over the long-term (3,4).

Observations have shown that healthy infants experience transient hypoglycemia as a part of the normal adaption to extrauterine life, with a decline in blood glucose concentrations to values as low as 20 to 25 mg/dL in the first two hours of life(4). Neonatal hypoglycemia persisting beyond 48 hours after birth might be prolonged transitional hypoglycemia(3).

Deviations from the normal transition can occur as a result of a wide range of factors predisposing individuals to neonatal hypoglycemia. The brain uses glucose primarily to meet its metabolic requirements. Therefore, neonatal hypoglycemia is a significant medical emergency in neonatal units. The signs and symptoms are often nonspecific. Some neonates are completely asymptomatic. Many cases, therefore, remain undiagnosed. The diagnosis of neonatal hypoglycemia requires a high index of suspicion since its presentation is different. Early diagnosis and treatment could prevent abrupt signs and symptoms, long-term complications, and death (2,5,6).

The neonatal hypoglycemia is still a severe concern because it causes neurological defect, an increased risk of mortality, and other complications in neonates and families. A study conducted by Shah R et al., (2019) revealed that the risk of a specific cognitive defect increased by 2-3-fold in early childhood. Neonatal hypoglycemia had odds of 3.2 for neurodevelopmental impairment and 2.04 for low literacy at school in late childhood (7). And, neonates with hypoglycemia would developed cerebral palsy at 8% in their childhood(8).

Moreover, a study in Tanzania showed that hypoglycemia was a major cause of neonatal mortality, contributing to 20% of all causes. Other family-related burdens secondary to neonatal hypoglycemia admission include increased costs to the family and separation from the mother(9). Thus, detection and treatment of these groups of neonates are targeted to reduce neonatal death, mainly in low-resource countries (10–13). According to some studies, neonatal hypoglycemia affects up to approximately 4-5 per 1000 term infants (5,6). A blood glucose level (RBS) below 47 mg/dL (2.6 mmol/L), if present during the 5 days during the first two months of life, was significantly correlated with acute seizure activity, abnormal neuromotor manifestations, and impaired intellectual performance at 18 months of age(14).

Despite progress over the past two decades, the age distribution of the mortality of children and young adolescents shows that the highest risk of death occurs during the neonatal period (the first 28 days of life), during which preventable causes accounted for 2.5 million out of 6.2 million deaths of children and young adolescents in 2018 alone(4,15). The neonatal mortality rate was estimated to be 18 deaths/1,000 live births globally(4,15). The probability of dying after the first month and before reaching age 1 was 11 per 1,000 live births (4,15).

According to the Ethiopian Demographic Health Survey (EDHS), Ethiopia has one of the highest neonatal mortality rates in the world (29 per 1000 live births), which represents poor progress in reducing the rate of neonatal mortality from 2000 to 2016(16). The report of previous studies on the prevalence of neonatal hypoglycemia and its risk factors were inconsistent in findings, and it is the first by its nature on prevalence of prolonged transitional hypoglycemia and associated factors in Ethiopia. The increased prevalence of prematurity and low birth weight make such a review vital for the formulation of prevention and management to neonates in specific and under-five children in general.

# Methods

Formatted: Font: 16 pt

The results of this review were reported based on the Preferred Reporting Items for Systematic Review and Meta-analysis (PRISMA) statement guidelines(17)(~~. ~~**Supplementary file 1)** ~~shows PRISMA Checklist. ~~The protocol is registered in the PROSPERO database (PROSPERO ID: CRD42023424953; https://www.crd.york.ac.uk/prospero/myprospero.

## Search strategy and information source

The adapted PICO format was used to explicitly review the literature and clarify the specifications of the inclusion and exclusion criteria. The adapted PICO comprises population (P), exposure (E), outcome (O), and context (setting as described below).

a. **Population**: all neonates irrespective of gestional age and birth weight
b. **Exposure**: Associated factors, predictors, risk factors
c. **Outcome**: prolonged transitional neonatal hypoglycemia
d. **Context (Setting):** Ethiopia.

Hence, by using this adapted PICO format, the following review questions were developed for the search for data.

1. What is the national incidence of prolonged transitional neonatal hypoglycemia in Ethiopia?
2. What are the factors associated with prolonged transitional neonatal hypoglycemia in Ethiopia?

Based on the aforementioned review PICO format and questions, primary studies were identified from MEDLINE, Scopus, Embase, Science Direct, Google Scholar, the African Journal Online, the Addis Ababa University repository, and the Haramay University repository.

The core search terms and phrases used were “prevalence”, “incidence”, “epidemiology”, “proportion”, “magnitude”, “burden”, “persistent”, “late newborn”, “recurrent”, “low blood glucose”, “predictors”, “risk factors”, “associated factors” and “Ethiopia”. The search strategies were developed using different Boolean operators. Notably, to fit the advanced MEDLINE database, the following search strategy was applied on April 8, 2023: [(prevalence[MeSH Terms]) OR incidence[MeSH Terms]) OR proportion [MeSH Terms]) OR epidemiology[MeSH Terms]) OR magnitude[MeSH Terms]) OR burden[MeSH Terms]) AND predictors [MeSH Terms]) OR risk factors [MeSH Terms]) OR associated factors [MeSH Terms]) OR neonatal hypoglycemia [MeSH Terms]) OR newborn hypoglycemia [MeSH Terms]) OR low blood

glucose in newborns [MeSH Terms]) OR low random blood glucose level in neonates [MeSH Terms]) AND (Ethiopia)].

### Study selection

Primary studies downloaded and retrieved were exported to Mendeley Desktop 1.19.8 reference manager software to remove duplicate studies. The study selection process was held in two stages. First, the title and abstract were screened, and second, a full-text review was performed. Two independent reviewers (DKM and SZB) screened the title and abstract. Disagreements were resolved based on established article selection criteria, and the third author (SDK) resolved disagreements to decide whether the study was eligible for inclusion if the quality appraisal score was 5 and more out of 9. Two independent authors subsequently reviewed the abstracts and full texts of the eligible articles.

## Eligibility criteria

**The inclusion criteria for patients were as follows**: articles that reported the prevalence of prolonged transitional neonatal hypoglycemia in general and/or at least one associated factor and published in English and studies conducted only in Ethiopia.

**The exclusion criteria for patients were as follows:** forms of hyperinsulinism or other diseases causing hypoglycemia such as inborn errors of metabolism were excluded. Moreover, articles without full-text information and qualitative studies were not reviewed.

## Critical appraisal and quality assessment

We used the quality appraisal criteria of the Joanna Briggs Institute (JBI)(18). The tool consists of nine major items. The first item is appropriate for the sample frame. The second is the appropriate sampling technique. The third is the adequacy of the sample size. The fourth is a description of the study subjects and settings. The fifth is enough coverage of the data analysis. The sixth is the validity of the method for identifying the condition. The seventh item is a standard and reliable measurement for all participants. The eighth is the appropriateness of the statistical analysis. The last item is adequacy and management of the response rate. Studies were considered low risk when they fit 5 or more quality assessment checklists. Two independent

**Formatted:** Font: 16 pt

**Formatted:** Font: 14 pt

**Formatted:** Font: 12 pt

**Formatted:** Font: 14 pt

**Formatted:** Font: 14 pt

**Formatted:** Font: 16 pt

authors (TMA and WNA) appraised the quality of the studies. Disagreements were resolved by the interference of a third reviewer (SDK). The quality scores for the primary studies were reported. **Supplementary file 2** shows JBI quality appraisal criteria.

## Data extraction

**Formatted:** Font: 16 pt

The data were determined to be extracted based on two criteria: 1. Clear and consistent operational definitions for the dependent variable (persistent neonatal hypoglycemia) and 2. These variables are statistically associated with the outcome variable reported by the AOR. Two authors extracted the data using the standardized format of the MS Excel spreadsheet **(Supplementary file 3)**. The name of the first author and year, study region, study area, study design, random blood glucose (RBS) cutoff point, sample size, prevalence, and odds of associated factors were extracted. Whenever variations were observed, the phase was repeated. If discrepancies between the data were resolved, a third reviewer was consulted. After reporting the inconsistency of the data in the primary study, data transformation was conducted.

## Outcome measures

**Formatted:** Font: 16 pt

**Prolonged transitional neonatal hypoglycemia (PTNHG):** Neonates with a random blood sugar (RBS) concentration <47 mg/dL at 48-72 hours after birth at the bedside by a random blood glucose analysis from a capillary sample (3,4).

**Formatted:** Font: 14 pt

## Statistical analysis

**Formatted:** Font: 16 pt

The required data were collected using the Microsoft Excel 2013 workbook. Then, STATA version 17 statistical software was used for the meta-analysis. Publication bias was objectively checked using Egger's regression test analysis(19). The heterogeneity of the studies was quantified using the I-squared statistic (20). Both pooled prevalence analysis and pooled effect of associated factors were conducted using a weighted inverse variance random-effects model (21). Subgroup analysis was performed by diagnostic method and sample size to overcome the inflation of the pooled effect from the inclusion of studies.

## Results

**Formatted:** Font: 16 pt

## Literature search:

The search strategy retrieved 64 articles electronically and manually, of which 18 duplicate articles were identified and removed. Among the 46 screened articles, 46 were from PubMed (n=19), Google Scholar (n=23), and other databases of the Addis Ababa University repository (n=2), the Haramaya University database (n=1), and the manuscript (n=1). The full texts of nineteen articles were reviewed, and eight studies were included in the review and meta-analysis. **Figure 1** shows an adapted PRISMA 2020 flow diagram for new systematic reviews that include searches of databases, registers, and other sources in Ethiopia.

## Characteristics of the included studies

**Formatted:** Font: 16 pt

Eight cross-sectional studies with a pooled sample size of 3,686 neonates were included in this meta-analysis. Two studies were found in each part of Ethiopia, namely, at Central, Eastern, Northwest and Northern (5,22–28). Four studies used an RBS cutoff of <40 mg/dl for the diagnosis of prolonged transitional neonatal hypoglycemia (22,23,27,28). The remaining four studies used an RBS cutoff point of less than 47 mg/dl for the diagnosis of PTNHGs (5,25,26,29). The maximum sample size was 769, and the minimum sample size was 196 among the reviewed studies (27,29). The studies included in this systematic review and meta-analysis had no considerable risk. Therefore, all the studies were considered. We assessed the studies with a JBI quality appraisal checklist for systematic review (30). According to this appraisal checklist, none of the included studies were of poor quality and had a quality score ≥6 (**Table 1).**

**Table 1.** Characteristics and quality status of the studies included, 2023.

**Formatted:** Left

| Author | Parts of Ethiopia | Capillary blood RBS cut point | Sample size | Incidence ~~Prevalence~~(%) of PNHG | JBI quality status |
|---|---|---|---|---|---|
| Bogale et al., 2021 | Northern (UOG) | <40 mg/dl | 399 | 13.53 | Low risk |
| Demis et al., 2022 | North-central | <47 mg/dl | 400 | 23.50 | Low risk |

| | | | | | |
|---|---|---|---|---|---|
| | (DTCSH) | | | | |
| Fantahun & Nurussen, 2021 | Central St. (PHMMC) | <47 mg/dl | 196 | 25.00 | Low risk |
| Kasaye, 2021 | Central (Addis Ababa) | <40 mg/dl | 317 | 20.82 | Low risk |
| Sertsu et al., 2022 | Eastern (HFSTH) | <40 mg/dl | 698 | 21.20 | Low risk |
| Yohannes, 2021 | Eastern (Harar and Dire Dawa) | <47 mg/dl | 640 | 14.68 | Low risk |
| Chanie et al., 2023 | North-central (DTCSH) | <47 mg/dl | 267 | 23.60 | Low risk |
| Demisse et al., 2017 | Northern (UOG) | <40 mg/dl | 769 | 18.46 | Low risk |

**UOG=University of Gondar, St. PHMMC=Saint Paul's Millennium Medical College, DTCSH=Debre Tabor Comprehensive Specialized Hospital**

## Meta-analysis

**Formatted:** Font: 16 pt

**Formatted:** Font: 11 pt

The pooled prevalence of prolonged transitional neonatal hypoglycemia was reviewed, and the absence of publication bias was assessed with Egger's regression test analysis (p = 0.068), which showed no publication bias (**Table 2**).

**Table 2**: Egger's test indicating no publication bias.

| Std.-Eff. | Coef. | Std. Err | t | p>t | 95% confidence interval | |
|---|---|---|---|---|---|---|
| **Slope** | 5.21962 | 2.177999 | 2.40 | 0.054 | -.1097508 | 10.54899 |
| **Bias** | 9.567008 | 4.312786 | 2.22 | **0.068** | -.9859986 | 20.12001 |

The pooled prevalence of PTNHG from eight studies (22–29) was 19.71 (95% CI: 16.85-22.56; $I^2$=79.20, P<0.001). **Figure 2** Forest plot for the pooled prevalence of PTNHG in Ethiopia, 2023.

## Subgroup analysis

**Formatted:** Font: 16 pt

Subgroup analysis was performed by using the sample size and the RBS cutoff point to diagnose prolonged transitional neonatal hypoglycemia (PTNHG). However, there was no effect on reducing the heterogeneity of the heterogeneity ($I^2$). According to the three studies of pooled effects, the prevalence with sample sizes >400 was 18% (15-22), and the prevalence with sample sizes ≤ 400 was 21% (17-26). The pooled effect of the RBS cutoff point for the diagnosis of PTNHG by (<40 mg/dl) were 18% (15-22), and by (<47 mg/dl) was 21% (16-27) (**Table 3)**.

**Table 3**. Subgroup analysis by sample size category and random blood sugar cutoff point for PTNHG and associated factors in Ethiopia, 2023.

| **Subgrouping by variables** | **Authors** | **Pooled prevalence at (95% CI)** | **$I^2$(P value)** |
|---|---|---|---|
| Sample size >400 | (23,24,27) | 18(15-22) | 78%(0.01) |
| Sample size ≤400 | (22,25,26,28,29) | 21(17-26) | 79.67%(0.00) |
| RBS<40 mg/dL | (22,23,27,28) | 18(15-22) | 73.83%(0.01) |
| RBS<47 mg/dL | (24–26,29) | 21(16-27) | 85.34%(0.00) |

## Factors associated with prolonged transitional neonatal hypoglycemia

Formatted: Font: 16 pt

Based on this review, the factors associated with persistent neonatal hypoglycemia identified by pooling two or more studies were preterm birth, neonatal hypothermia, neonatal birth from a chronic diabetic mellitus mother, breastfeeding initiation after an hour, and pathological jaundice.

According to previous studies (23,26), neonates who were delivered prematurely were three more likely to develop prolonged transitional neonatal hypoglycemia than neonates who were born at term in Ethiopia (AOR=3.31; 95% CI=2.57-4.04)(~~. **Figure 3** shows the pooled effect of preterm birth as a factor associated with PTNHG in Ethiopia.~~

A pooled analysis of four studies (23,25,26,29) revealed that neonates with hypothermia were 3.41 times more likely (AOR=3.41; 95% CI=2.19-4.62) to develop PTNHG than their normotensive counterparts were(~~. **Figure 4** shows the pooled effect of hypothermia as a factor associated with PTNHG in Ethiopia.~~

There were nearly five times more neonates whose mothers had known chronic DM (AOR=4.71; 95% CI=2.15-7.26) than did those whose mothers did not have known chronic DM (22,23,25)(~~. **Figure 5** shows the pooled effect of chronic diabetes mother as a factor associated with PTNHG in Ethiopia~~.

According to previous studies (22,23), neonates who had been breastfeeding after an hour were three times more likely to be hypoglycemic than neonates who had been breastfeeding within an hour (AOR=3.26; 95% CI=2.03-4.49)(~~. **Figure 6** shows the pooled effect of breastfeeding after an hour as factor associated with PTNHG in Ethiopia.~~

~~-~~Pooling of two studies(24,26) revealed that the incidence of pathological jaundice in neonates was almost twofold-fold greater (AOR=1.70; 95% CI=1.21-2.99) than that in their counterparts

without pathological jaundice~~(. **Figure 7** shows the pooled effect of pathological jaundice as a factor associated with PTNHG in Ethiopia.~~

**Table 4**: The pooled effect of factors associated with PNHG in Ethiopia, 2023.

| **Factors** | **Author and year** | **Pooled OR (95% CI)** | **$I^2$(P value)** |
|---|---|---|---|
| Preterm birth | (Sertsu *et al.*, 2022) | 3.31(2.57-4.04) | 0.01%(0.001) |
| | (Demis *et al.*, 2022) | | |
| Neonatal Hypothermia | (Sertsu *et al.*, 2022) | 3.41(2.19-4.62) | 97.96%(0.001) |
| | (Fantahun and Nurussen, 2021) | | |
| | (Chanie *et al.*, 2023) | | |
| | (Demis *et al.*, 2022) | | |
| Infants of diabetic mother (IDM) | (Sertsu *et al.*, 2022) | 4.71(2.15-7.26) | 94%(0.001) |
| | (Chanie *et al.*, 2023) | | |
| | (Kasaye et al., 2021) | | |
| Breastfeeding after one hour | (Sertsu *et al.*, 2022) | 3.26(2.03-4.49) | 52.80%(0.001) |
| | (Kasaye et al., 2021) | | |
| Pathological jaundice | (Demis *et al.*, 2022) | 2.37(1.91-2.84) | 0.01%(0.001) |
| | (Yohannes et al., 2021) | | |

**Data extracted date was on 20-27/05/2023, Data extractors were SDK, TMA, DK and KA**

One study (23) revealed that the odds of neonatal sepsis were nearly three times greater than that of neonates with no sepsis, and the odds of neonates with very low birth weight (VLBW) were four times greater than those of neonates with normal birth weight(NBW) (AOR=2.61; 95% CI=1.68-3.54) and (AOR=4.01; 95% CI=1.85-9.87), respectively. According to a previous study (25), for neonates with one or more episodes of seizure and asphyxia stage II or III HIE, PTNHG had odds of nearly five times and five times greater than their counterparts (AOR=4.70; 95% CI=3.78-5.62) and (AOR=5.10; 95% CI=4.18-6.02), respectively. Other single studies (Kasaye, 2021) showed that neonates whose mothers had GDM or preeclampsia during their latest pregnancy were approximately eight or two times more likely to have GDM or preeclampsia, respectively (AOR=7.79; 95% CI=5.87-9.71) and (AOR=2.44; 95% CI=1.65-3.23), than their counterparts were. In contrast, a study by (26) revealed that neonates delivered via SVD were 28~~72~~% less likely to develop PTNHG than were those delivered via CS (AOR=0.72; ~~-~~0.35-1.79).

# Discussion

Formatted: Font: 16 pt

This systematic review and meta-analysis aimed to assess the pooled incidence of PTNHGs and associated factors in Ethiopia. The pooled prevalence was 19.71 (95% CI: 16.85-22.56), which was comparable to that reported in studies in Iran Tehran (31), China Shanghai (32) and the USA (33), which had a prevalence of 15.50% and 16.90%, respectively, and a range of 8-30%, respectively.

On the other hand, studies from Pakistan used RBS cutoff values <50 mg/dl (34), and on the basis of RBS cutoff values <40 mg/dl and 47 mg/dl (35), it was found that the incidence of neonatal hypoglycemia was much lower than that in this review (0.4% and 3.4-12.10%, respectively). This might be because this review utilized the operational definition of persistent neonatal hypoglycemia, and the aforementioned literature included all types of neonatal hypoglycemia. Moreover, the variation in sample size and socio-demographic variables might contribute to this difference.

According to our subgroup analysis based on the RBS cutoff for diagnosing PTNHG, the estimated pooled prevalence obtained by pooling the studies with an RBS cutoff<47 mg/dl was 21% greater (95% CI: 16-27) than that obtained by pooling the studies with an RBS cutoff <40 mg/dl 18% (95% CI: 15-22). A possible explanation might be the difference between the RBS cutoff points; therefore, <47 mg/dl is associated with a greater probability that more neonates would have RBS measurements within that range. This result is supported by studies in Israel in which the incidence of neonatal hypoglycemia substantially differed between the RBS cutoff <40 mg/dl and <47 mg/dl at 3.4% and 12.10%, respectively (35). Similarly, there was a difference in the pooled estimate of PTNHG between the primary studies with a sample size >400 at 18% (95% CI: 15-22) and those with ≤400 at 21% (95% CI: 17-26). This difference can be explained by the fact that studies with sample size variation affected the outcome of the data analysis. Hence, a sample size >400 had more strength in determining the true value statistically in the regression analysis than a sample size ≤400.

This meta-analysis revealed that preterm infants were 3 times more likely to develop PTNHG than term neonates were. This could be because preterm neonates are uniquely predisposed to hypoglycemia of any type and its related complications due to their limited glycogen and fat

stores, through which body thermoregulation can be maintained. Premature neonates' inability to generate new glucose via gluconeogenesis pathways affects their glucose utilization in metabolic processes. This, in turn, requires greater metabolic demands due to having a relatively larger brain than term neonates because they are unable to respond to a counter regulatory mechanism to hypoglycemia compared to term neonates (23,36,37).

Similarly, another factor associated with PTNHGs was neonatal hypothermia, which increased the odds of PTNHG development by 3-fold compared with that of their counterparts with normothermic neonates. The scientific evidence was congruent with these findings in that neonatal hypothermia predisposes to neonatal hypoglycemia. Neonates exhibit a metabolic response to cooling that involves chemical but no shivering thermogenesis via the sympathetic nerve discharge of norepinephrine in brown fat. This reaction increases the metabolic rate and oxygen consumption 2-3-fold by activating glycogen stores, thereby causing transient hypoglycemia as a compensatory mechanism and persistent hypoglycemia as a disorder, as impaired body glucose hemostasis results in metabolic acidosis and increased risk of death (38–42).

Neonates who gave birth from mothers with chronic diabetes mellitus were nearly five times more likely to develop PTNHG than were those from mothers without chronic diabetes mellitus. The justification for this finding is that an infant of a diabetic mother (IDM) is more likely to have a period of low blood sugar shortly after birth and during the first few days of life.

This might be because neonates obtain more sugar than needed from the mother in the uterus before delivery. As such, neonates have higher insulin levels than needed after birth, and insulin then lowers their blood sugar level, allowing them to adjust to a normal RBS after birth for several days (43–45).

Regarding breastfeeding initiation time, the odds that neonates who initiated breastfeeding after an hour were approximately three times greater (AOR=3.26; 95% CI=2.03-4.49) of developing PTNHG than were those who breastfed within an hour after delivery (22,23). This evidence, combined with the pooled ORs estimated, was strongly reflected in the results of a single study indicating that neonates who delivered via spontaneous vaginal delivery(SVD) were 72% less likely (AOR=0.72; -0.35-1.79) to develop PTNHG than were those who delivered via cesarean section delivery (CS) (26). This is justified by the fact that breast milk is the ideal food for

infants that protects against neonatal hypoglycemia. The initiation of breastfeeding within an hour is the mainstay for preventing persistent neonatal hypoglycemia, as per WHO recommendations. However, neonates who delivered via the CS were 2.01 times more likely to develop neonatal hypoglycemia. Hence, any cause that delays breastfeeding within one hour, including CS delivery, should be considered. A delay in breastfeeding results in neonatal hypoglycemia because breast milk provides all the energy and nutrients that the infant needs for the first months of life and continues to provide up to half or more of a child's nutritional needs (1,46–48).

On the other hand, neonates with pathological jaundice had approximately 2.37 greater odds (AOR=2.37; 95% CI=1.91-2.84) than did those without pathological jaundice (24,26). These findings are comparable to those of other studies in that neonates with hyper-bilirubinemia were 1.81 and 1.93 times more likely to develop neonatal hypoglycemia than were those with normal bilirubin test values. In another study, 8.80% of neonates treated with phototherapy developed neonatal hypoglycemia (48–50).

This might be because the relationship between neonatal hypoglycemia and neonatal hyper-bilirubinemia in pathological jaundice might be mutually influenced by the fact that OGTT 2-h glucose levels affect neonatal hypoglycemia. Abnormal postprandial glucose levels suggested impaired glucose tolerance and β-cell dysfunction on a physiological basis due to jaundice. As a result, neonates with jaundice are affected by the physiological process of gluconeogenesis to maintain a norm-glycemic state.

According to the findings from a single primary study, patients diagnosed with early neonatal sepsis had greater odds of developing neonatal hypoglycemia (AOR=2.61; 95% CI=1.68-3.54). These findings were supported by the findings of the literature that early onset of neonatal sepsis (EONS) with neonatal hypoglycemia was approximately 8.7% and 9.6%, respectively, among neonates admitted for sepsis.

This is because neonates with neonatal sepsis tend to refuse to breastfeed, resulting in low blood sugar. Similarly, increased metabolic demand is caused by low glucose levels in sepsis and hypothermia (51,52). According to a previous study (25), for neonates with one or more episodes of seizure and asphyxia stage II or III HIE, PTNHG had odds of nearly five times and five times greater than their counterparts (AOR=4.70; 95% CI=3.78-5.62) and (AOR=5.10; 95% CI=4.18-

6.02), respectively. This increased risk for hypoglycemia might be due to a lack of storage in combination with high insulin levels, which predisposes patients to hypoglycemia. Perinatal asphyxia (PNA) and perinatal seizures secondary to PNA increase the risk of hyper-insulinism in the neonatal period because of the use of anaerobic metabolism to maintain blood glucose concentrations. Anaerobic metabolism is used to maintain blood glucose concentrations (53). Finally, a study (22) showed that neonates with gestational diabetes mellitus (GDM) or preeclampsia during their latest pregnancy were approximately eight times and two times more likely, respectively (AOR=7.79; 95% CI=5.87-9.71) and (AOR=2.44; 95% CI=1.65-3.23), than their counterparts were. This can be explained by the fact that the most common complication in infants born to GDM mothers is hypoglycemia. This is due to glucose freely passing through the placenta; therefore, maternal hyperglycemia associated with GDM results in elevated glucose levels in the fetus, causing excess fetal insulin production, termed hyper-insulinism(54).

# Conclusion

**Formatted:** Font: 16 pt

Persistent neonatal hypoglycemia is quite common in Ethiopia. The pooled prevalence estimate of PTNHG from eight studies was nearly ten percent. Neonates who delivered prematurely, neonates with hypothermia, neonates of diabetic mothers (IDM), neonates who had not been breastfeeding after an hour and neonates with pathological jaundice had higher odds of developing PNHG than did their counterparts. According to single primary studies, neonatal sepsis, very low birth weight(VLBW), neonates with one or more episodes of seizure, and asphyxia stage II or III HIE, neonates of GDM mothers had higher odds of developing neonatal hypoglycemia. In contrast, neonates delivered by SVD were protected against neonatal hypoglycemia. As per this review, the pooled prevalence of PNHGs was significant. Several associated factors were pooled so that intervention strategies would be needed to reduce and prevent their occurrence while treating neonates in neonatal intensive care units in Ethiopia. Thus, the team of this review strongly advised health care professionals, including nurses, doctors, and others working in the NICU, to monitor and regulate these and other determining variables that predispose patients to neonatal hypoglycemia, particularly the persistent type.

## Contribution of the Review

Given the national prevalence and associated factors of persistent neonatal hypoglycemia (PNHG), evidence-based decisions will be used in the prevention and treatment of persistent neonatal hypoglycemia in health care settings. Hence, healthcare professionals, others who work in the neonatal intensive care unit (NICU) and the directors of those services can adopt this finding by incorporating it as the priority concern in neonatal care standards in Ethiopia.

## Availability of data

The data was uploaded as supplementary information.

**Formatted:** Font: (Default) Times New Roman, 12 pt

## Ethics approval

Not applicable.

## Conflicts of interest

All authors declared no conflicts of interests

## Author contributions

**Conceptualization:** Solomon D. Kebede

**Data curation:** Kindu Agmas

**Formal analysis and methodology:** Solomon D. Kebede

**Supervision and commenting:** Demewoz Kefale and Amare Kassaw

**Writing original draft:** Solomon D. Kebede and Tigabu M. Aytenew

**Writing, review and editing:** Solomon D. Kebede

## Funding

**The authors received no specific funding for this work**

## Patient and public involvement

Patient and/or the public were not involved in the design, or conduct, or reporting, or dissemination plans of this research.

**Abbreviations:** **AOR**-Adjusted Odds Ratio, **CI**-Confidence Interval, **DRC**-Democratic Republic of Congo, **MCH**-Maternal Child Health, **TCU**-Traditional Childhood Uvulectomy, **WHO**-World Health Organization.

## Abbreviations

**CS**: Cesarean Section, **GDM**: Gestational Debates Mellitus, **HIE**: Hypoxic Ischemic Encephalopathy, **IDM**: Infant of Diabetics' Mother, **PNHG:** Persistent Neonatal Hypoglycemia, **SGA**: Small for Gestational Age, **SVD**: Spontaneous Vaginal Delivery, **VLBW**: Very Low Birth Weight

**Formatted:** Indent: Left: 0", First line: 0"

# References

**Formatted:** Font: 16 pt


1. WHO. Guideline:Protecting, promoting and supporting breastfeeding in facilities providing maternity and newborn services [Internet]. World Health OrganizationWHO. 2017. 1–136 p. Available from: https://apps.who.int/iris/bitstream/handle/10665/259386/9789241550086-eng.pdf

2. Cornblath M, Hawdon JM, Williams AF, Aynsley-Green A, Ward-Platt MP, Schwartz R, et al. Controversies regarding definition of neonatal hypoglycemia: Suggested operational thresholds. Pediatrics. 2000;105(5):1141–5.

3. Thompson-Branch A, Havranek T. Neonatal hypoglycemia. Pediatr Rev. 2017;38(4):147–57.

4. Abramowski A, Ward R, Hamdan AH. Neonatal hypoglycemia. In: StatPearls [Internet]. StatPearls Publishing; 2021.

5. Paul S, Yismaw Fantahun B. Prevalence and Risk factors of Hypoglycaemia in Neonates at: A Cross Sectional Study. 2020;1–15.

6. Frank-Briggs AI, Ojule AC, Nkanginieme KE. Neonatal hypoglycaemia: prevalence and clinical manifestations in Port Harcourt, Nigeria. Port Harcourt Med J. 2008;2(2):166–70.

7. Shah R, Harding J, Brown J, McKinlay C. Neonatal glycaemia and neurodevelopmental outcomes: a systematic review and meta-analysis. Neonatology. 2019;115(2):116–26.

8. Mahajan G, Mukhopadhyay K, Attri S, Kumar P. Neurodevelopmental Outcome of Asymptomatic Hypoglycemia Compared With Symptomatic Hypoglycemia and Euglycemia in High-Risk Neonates. Pediatr Neurol [Internet]. 2017;74:74–9. Available from: https://www.sciencedirect.com/science/article/pii/S0887899417302825

9. Sultan OM. The Hidden Burden of Hypoglycemia in Neonatal Mortality in Tanzania-The Case of Saint Francis Hospital, Ifakara. IMTU Med J. 2012;3:34–9.

10. Kush ML, Baschat AA. Fetal responses to placental insufficiency. In: Textbook of Perinatal Medicine. CRC Press; 2006. p. 1021–38.

11. Menni F, De Lonlay P, Sevin C, Touati G, Peigné C, Barbier V, et al. Neurologic outcomes of 90 neonates and infants with persistent hyperinsulinemic hypoglycemia. Pediatrics. 2001;107(3):476–9.

12. Tam EWY, Widjaja E, Blaser SI, MacGregor DL, Satodia P, Moore AM. Occipital lobe injury and cortical visual outcomes after neonatal hypoglycemia. Pediatrics. 2008;122(3):507–12.

13. McKinlay CJD, Alsweiler JM, Anstice NS, Burakevych N, Chakraborty A, Chase JG, et al. Association of neonatal glycemia with neurodevelopmental outcomes at 4.5 years. JAMA Pediatr. 2017;171(10):972–83.

14. Lucas A, Morley R, Cole TJ. Adverse neurodevelopmental outcome of moderate neonatal hypoglycaemia. BMJ. 1988 Nov;297(6659):1304–8.

15. You D, Hug L, Ejdemyr S, Idele P, Hogan D, Mathers C, et al. Global, regional, and national levels and trends in under-5 mortality between 1990 and 2015, with scenario-based projections to 2030: a systematic analysis by the UN Inter-agency Group for Child Mortality Estimation. Lancet. 2015;386(10010):2275–86.

16. Gebremichael SG, Fenta SM. Under-Five Mortality and Associated Risk Factors in Rural Settings of Ethiopia: Evidences from 2016 Ethiopian Demographic and Health Survey. Adv Public Heal. 2020;2020.

17. Moher D, Shamseer L, Clarke M, Ghersi D, Liberati A, Petticrew M, et al. Preferred reporting items for systematic review and meta-analysis protocols (PRISMA-P) 2015 statement. Syst Rev. 2015;4(1):1–9.

18. Peters MDJ, Godfrey CM, McInerney P, Soares CB, Khalil H, Parker D. The Joanna Briggs Institute reviewers' manual 2015: methodology for JBI scoping reviews. 2015;

19. Rothstein HR, Sutton AJ, Borenstein M. Publication bias in meta-analysis. Publ bias meta-analysis Prev Assess Adjust. 2005;1–7.

20. Ioannidis JPA. Interpretation of tests of heterogeneity and bias in meta-analysis. J Eval Clin Pract. 2008;14(5):951–7.

21. Borenstein M, Hedges L V, Higgins JPT, Rothstein HR. A basic introduction to fixed-effect and random-effects models for meta-analysis. Res Synth Methods. 2010;1(2):97–111.

22. KASAYE F. COLLEGE OF HEALTH SCIENCE SCHOOL OF NURSING AND MIDWIFERY DEPARTMENT OF NURSING PREVALENCE AND PREDICTORS OF HYPOGLYCEMIA AMONG NEONATES ON ADMISSION TO NEONATAL INTENSIVE CARE UNIT IN SELECTED PUBLIC HOSPITALS , PRINCIPAL INVESTIGATOR : FIKIRTE KASAYE (. 2021;

23. Sertsu A, Nigussie K, Eyeberu A, Tibebu A, Negash A, Getachew T, et al. Determinants of neonatal hypoglycemia among neonates admitted at Hiwot Fana Comprehensive Specialized University Hospital, Eastern Ethiopia: A retrospective cross-sectional study. SAGE Open Med. 2022;10.

24. YOHANNES M. Magnitude of Jaundice and Associated Factors among Neonates Admitted to Neonatal In_tensive Care Unit in Eastern Ethiopia. Front Neurosci. 2021;14(1):1–13.

25. Chanie ES, Shiferaw SM, Feleke DG, Bantie B, Moges N, Tasew SF, et al. Maximizing the detection rate of hypoglycemia among preterm neonates admitted in Neonatal intensive care unit in Ethiopia, 2021. Sci Rep [Internet]. 2023;13(1):1–7. Available from: https://doi.org/10.1038/s41598-023-29112-y

26. Demis S, Munye T, Agmas K, Necho W, Moges N. Incidence of Persistent Neonatal Hypoglycemia and Associated Factors among Neonatal Intensive Care Unit Admissions in South Gondar Public Hospitals , Northcentral Ethiopia : a prospective cross sectional study. unpublished. 2022;1–24.

27. Demisse AG, Alemu F, Gizaw MA, Tigabu Z. Patterns of admission and factors associated with neonatal mortality among neonates admitted to the neonatal intensive care unit of University of Gondar Hospital, Northwest Ethiopia. Pediatr Heal Med Ther. 2017;Volume 8:57–64.

28. Bogale MT, Bogale WA, Kassie DG, Woldesellassie A, Tamiru AT. Factors associated with neonatal hyperbilirubinemia in case files of all admitted inborn and outborn neonates in Northwest Ethiopia in 2019. Iran J Neonatol. 2021;12(1):46–53.

29. Fantahun B, Nurussen I. Prevalence and risk factors of neonatal hypoglycaemia at st. Paul’s Hospital Millennium Medical College, Ethiopia. Pediatr Child Heal [Internet]. 2021;26(1):13. Available from: https://www.researchgate.net/profile/Bereket-Fantahun-2/publication/358834078_ORIGINAL_ARTICLE_PREVALENCE_AND_RISK_FACTORS_OF_NEONATAL_HYPOGLYCAEMIA_AT_ST_PAUL’S_HOSPITAL_MILLENNIUM_MEDICAL_COLLEGE_ETHIOPIA/links/6217e57e6738db292ac385ac/ORIGINAL-ARTICLE-

30. Briggs J. Checklist for Systematic Reviews and Research Syntheses [Internet]. The Joanna Briggs Institute. 2017. p. 7. Available from: http://joannabriggs.org/research/critical-appraisal-tools.htmlwww.joannabriggs.org%0Awww.joannabriggs.org

31. Dashti N, Einollahi N, Abbasi S. NEONATAL HYPOGLYCEMIA : PREVALENCE AND CLINICAL MANIFESTATIONS IN TEHRAN CHILDREN ' S HOSPITAL. 2007;23(3).

32. Zhou W, Yu J, Wu Y, Zhang H. Hypoglycemia incidence and risk factors assessment in hospitalized neonates neonates. 2015;7058:6–10.

33. Alemu BT, Olayinka O, Baydoun HA, Hoch M, Elci MA. Neonatal Hypoglycemia in Diabetic Mothers : a Systematic Review. 2017;21:42–53.

34. Najati N, Saboktakin L. Prevalence and underlying etiologies of neonatal hypoglycemia. Vol. 13, Pakistan Journal of Biological Sciences. 2010. p. 753–6.

35. Bromiker R, Perry A, Kasirer Y, Einav S, Klinger G. Early neonatal hypoglycemia : incidence of and risk factors . A cohort study using universal point of care screening. J Matern Neonatal Med [Internet]. 2019;32(5):786–92. Available from: https://doi.org/10.1080/14767058.2017.1391781

36. Sharma A, Davis A, Shekhawat PS. Hypoglycemia in the preterm neonate: etiopathogenesis, diagnosis, management and long-term outcomes. Transl Pediatr. 2017 Oct;6(4):335–48.

37. Goode RH, Rettiganti M, Li J, Lyle RE, Whiteside-Mansell L, Barrett KW, et al. Developmental outcomes of preterm infants with neonatal hypoglycemia. Pediatrics. 2016;138(6).

38. Yitayew YA, Aitaye EB, Lechissa HW, Gebeyehu LO. Neonatal Hypothermia and Associated Factors among Newborns Admitted in the Neonatal Intensive Care Unit of Dessie Referral Hospital, Amhara Region, Northeast Ethiopia. Int J Pediatr. 2020;2020:3013427.

39. Strauch, Barry S.Felig, PhilipBaxter JD. 2023. 2023.

40. Agardh CD, Smith ML, Siesjö BK. The influence of hypothermia on hypoglycemia-induced brain damage in the rat. Acta Neuropathol. 1992;83(4):379–85.

41. Kedes LH, Field JB. Hypothermia. N Engl J Med [Internet]. 1964 Oct 8;271(15):785–7. Available from: https://doi.org/10.1056/NEJM196410082711509

42. Tran C, Gariani K, Herrmann FR, Juan L, Philippe J, Rutschmann OT, et al. Hypothermia is a frequent sign of severe hypoglycaemia in patients with diabetes. Diabetes Metab. 2012 Oct;38(4):370–2.

43. Kallem VR, Pandita A, Pillai A. Infant of diabetic mother: what one needs to know? J Matern Neonatal Med [Internet]. 2020;33(3):482–92. Available from: https://doi.org/10.1080/14767058.2018.1494710

44. Mormile R. Neonates of diabetic mothers: The starting point for developing novel therapeutic approaches to ischemic heart and brain? Med Hypotheses. 2016 Nov;96:75–7.

45. Opara PI, Jaja T, Onubogu UC. Morbidity and mortality amongst infants of diabetic mothers admitted into a special care baby unit in Port Harcourt, Nigeria. Ital J Pediatr. 2010;36(1):2–7.

46. Ekubay M, Berhe A, Yisma E. Initiation of breastfeeding within one hour of birth among mothers with infants younger than or equal to 6 months of age attending public health institutions in Addis Ababa, Ethiopia. Int Breastfeed J. 2018;13(1):1–7.

47. Karim F, Masum Billah SK, Chowdhury MAK, Zaka N, Manu A, Arifeen S El, et al. Initiation of breastfeeding within one hour of birth and its determinants among normal vaginal deliveries at primary and secondary health facilities in Bangladesh: A case-observation study. PLoS One. 2018;13(8):1–15.

48. He J, Song J, Zou Z, Fan X, Tian R, Xu J, et al. Association between neonatal hyperbilirubinemia and hypoglycemia in Chinese women with diabetes in pregnancy and influence factors. Sci Rep [Internet]. 2022;12(1):16975. Available from: https://doi.org/10.1038/s41598-022-21114-6

49. Kallem VR, Pandita A, Pillai A. Infant of diabetic mother: what one needs to know? J Matern neonatal Med Off J Eur Assoc Perinat Med Fed Asia Ocean Perinat Soc Int Soc Perinat Obstet. 2020 Feb;33(3):482–92.

50. Salman M, Rathore H, Arif S, Ali R, Khan AA, Nasir M. Frequency of Immediate Neonatal Complications (Hypoglycemia and Neonatal Jaundice) in Late Preterm and Term Neonates. Cureus. 2021 Jan;13(1):e12512.

51. Islam MS, Mia MAH, Akhter KR, Haque M, Malik M. Glycemic Status and its Effect in Neonatal Sepsis in a Tertiary Care Hospital. Bangladesh J Child Heal. 2017;40(1):21–5.

52. Ellahony D, El-Mekkawy M, A. Issa AHR. Prevalence of glucose level abnormalities in neonatal sepsis and its association with mortality. Menoufia Med J. 2020;33(1):127.

53. Sweet CB, Grayson S, Polak M. Management strategies for neonatal hypoglycemia. J Pediatr Pharmacol Ther JPPT Off J PPAG. 2013 Jul;18(3):199–208.

54. Voormolen DN, de Wit L, van Rijn BB, DeVries JH, Heringa MP, Franx A, et al. Neonatal Hypoglycemia Following Diet-Controlled and Insulin-Treated Gestational Diabetes Mellitus. Diabetes Care [Internet]. 2018 Apr 13;41(7):1385–90. Available from: https://doi.org/10.2337/dc18-0048

## Supporting information

**S1 Supplementary 1**. PRISMA checklist

**S2 Supplementary file 2**. JBI quality appraisal criteria

**S3 Supplementary file 3.** Data of the review and meta-analysis

## Authors' response to reviewers' comments

"Prevalence of persistent neonatal hypoglycemia and its associated factors in Ethiopia: A systematic review and meta-analysis"

Good beginning; yet few areas of improvement and the readers/reviewers concerns / suggestion to be incorporated

1. Good title to capture the reader of different healthcare professionals
2. In the abstract it is mentioned; the sample size was 3686 from the 8 studies but later on it is mentioned 3600? It needs to be clear for the readers

   **Authors' response:** revised
3. Interested background and introduction; keeps the readers indulged in the reading; very good
4. Good rationale for the study and gives the readers novelty
5. PICO format has the biggest concerns: Readers are totally confused at once; sudden change from the hypoglycemia to intra-Ventricular hemorrhage???? Does that align with the title of the study? Turning point from the winners to losers in the eyes of the readers? Unbearable as reader is not happy with the twist; clear the reader what the study is about?

   **Authors' response**: revised and we ask for pardon. We made writing errors.
6. Completely opposing from the title as per readers best judgment??? Derailed from the track? I don't know how and why it happened??? Many questions arise. As stated in PICO format, (the following review questions were developed for the search for data. What is the magnitude of IVH in African countries? What are the predictors of IVH in African countries?) Reader is confused, what the outcome is stated and what it should be as per the title of the study???

   **Authors' response:** yes we did it correct.
7. The key terms in the abstract are mentioned as "Prevalence, persistent neonatal hypoglycemia, prolonged neonatal hypoglycemia, Ethiopia (these were the key words as stated earlier in the abstract); suddenly all these are mentioned later on as "The core search terms and phrases were "prevalence", "incidence", "epidemiology", "proportion", "magnitude", "burden", "Intraventricular" and ''predictors'', "risk factors'', ''associated factors'' and ''Africa'' Or ''African countries"???? Huge gap exists

**Authors' response**: corrected typos errors

8. time frame in the inclusion criteria need to be mentioned

   **Authors' response**: the articles included were those published from 2020 to 2022.

9. Pakistan; Iran and Israel are from different region (apart from Ethiopia); do these studies align with the inclusion criteria??? Referring to the second Para of the discussion chapter?

   **Authors' response**: this is to insight the burden across the globe…not to analyze It is aimed to show the difference in different regions but not part for this review.

10. Referring to Critical appraisal and quality assessment portion ; it would be more appropriate if the examples be given how the criteria was accomplished in the particular study?

    **Authors' response:** yes the comment is accepted and given full reference to supplementary file 2.

11. Strength and limitation of this study depicts 3600 neonates were included??? Does that mean a sample size??? If yes, previously it was mentioned that 3686 neonates were the ample size? Clarity is suggested

    **Authors' response**: revised and sorry for repeated writing errors.
