# Supplementary material for: Prevalence of prolonged transitional neonatal hypoglycemia and associated factors in Ethiopia: A systematic review and meta-analysis
Source: PLoS One. 2025 Feb 6;20(2):e0316464. doi: 10.1371/journal.pone.0316464 (PMC11801580; doi:10.1371/journal.pone.0316464)
Supplement: Attachment — Submitted filename: Responses to reviewers comments.docx [file pone.0316464.s007.docx]

**Authors’ response to reviewers’ comments**

“Prevalence of persistent neonatal hypoglycemia and its associated factors in Ethiopia: A systematic review and meta-analysis”

Good beginning; yet few areas of improvement and the readers/reviewers concerns / suggestion to be incorporated

1. Good title to capture the reader of different healthcare professionals
2. In the abstract it is mentioned; the sample size was 3686 from the 8 studies but later on it is mentioned 3600? It needs to be clear for the readers

**Authors’ response:** revised

3. Interested background and introduction; keeps the readers indulged in the reading; very good
4. Good rationale for the study and gives the readers novelty
5. PICO format has the biggest concerns: Readers are totally confused at once; sudden change from the hypoglycemia to intra-Ventricular hemorrhage???? Does that align with the title of the study? Turning point from the winners to losers in the eyes of the readers? Unbearable as reader is not happy with the twist; clear the reader what the study is about?

**Authors’ response**: revised and we ask for pardon. We made writing errors.

6. Completely opposing from the title as per readers best judgment??? Derailed from the track? I don't know how and why it happened??? Many questions arise. As stated in PICO format, (the following review questions were developed for the search for data. What is the magnitude of IVH in African countries? What are the predictors of IVH in African countries?) Reader is confused, what the outcome is stated and what it should be as per the title of the study???

**Authors’ response:** yes we did it correct.

7. The key terms in the abstract are mentioned as “Prevalence, persistent neonatal hypoglycemia, prolonged neonatal hypoglycemia, Ethiopia (these were the key words as stated earlier in the abstract); suddenly all these are mentioned later on as "The core search terms and phrases were “prevalence”, “incidence”, “epidemiology”, “proportion”, “magnitude”, “burden”, “Intraventricular” and ‘’predictors’’, “risk factors’’, ‘’associated factors’’ and ‘’Africa’’ Or ‘’African countries"???? Huge gap exists

**Authors’ response**: corrected typos errors

8. time frame in the inclusion criteria need to be mentioned

**Authors’ response**: the articles included were those published from 2020 to 2022.

9. Pakistan; Iran and Israel are from different region (apart from Ethiopia); do these studies align with the inclusion criteria??? Referring to the second Para of the discussion chapter?

**Authors’ response**: this is to insight the burden across the globe…not to analyze It is aimed to show the difference in different regions but not part for this review.

10. Referring to Critical appraisal and quality assessment portion ; it would be more appropriate if the examples be given how the criteria was accomplished in the particular study?

**Authors’ response:** yes the comment is accepted and given full reference to supplementary file 2.

11. Strength and limitation of this study depicts 3600 neonates were included??? Does that mean a sample size??? If yes, previously it was mentioned that 3686 neonates were the ample size? Clarity is suggested

**Authors’ response**: revised and sorry for repeated writing errors.
